# Drug repurposing against SARS-CoV-1, SARS-CoV-2 and MERS-CoV

**DOI:** 10.2217/fmb-2021-0019

**Published:** 2021-11-10

**Authors:** Sarah Aherfi, Bruno Pradines, Christian Devaux, Stéphane Honore, Philippe Colson, Bernard La Scola, Didier Raoult

**Affiliations:** ^1^Aix-Marseille Université, Assistance Publique – Hôpitaux de Marseille (AP-HM), Marseille, 13005, France; ^2^Institut Hospitalo-Universitaire (IHU) – Méditerranée Infection, Marseille, 13005, France; ^3^Microbes, Evolution, Phylogeny & Infection (MEΦI), Marseille, 13005, France; ^4^Unité Parasitologie et Entomologie, Département Microbiologie et Maladies Infectieuses, Institut de Recherche Biomédicale des Armées, Marseille, 13005, France; ^5^Aix-Marseille Univ, IRD, SSA, AP-HM, VITROME, Marseille, 13005, France; ^6^Centre national de référence du paludisme, Marseille, 13005, France; ^7^Aix Marseille Université, Laboratoire de Pharmacie Clinique, Marseille, 13005, France; ^8^AP-HM, hôpital Timone, service pharmacie, Marseille, 13005, France

**Keywords:** antiviral, coronavirus, COVID-19, drug repurposing, *in vitro* testing, *in silico*, SARS-CoV-1, SARS-CoV-2

## Abstract

Since the beginning of the COVID-19 pandemic, large *in silico* screening studies and numerous *in vitro* studies have assessed the antiviral activity of various drugs on SARS-CoV-2. In the context of health emergency, drug repurposing represents the most relevant strategy because of the reduced time for approval by international medicines agencies, the low cost of development and the well-known toxicity profile of such drugs. Herein, we aim to review drugs with *in vitro* antiviral activity against SARS-CoV-2, combined with molecular docking data and results from preliminary clinical studies. Finally, when considering all these previous findings, as well as the possibility of oral administration, 11 molecules consisting of nelfinavir, favipiravir, azithromycin, clofoctol, clofazimine, ivermectin, nitazoxanide, amodiaquine, heparin, chloroquine and hydroxychloroquine, show an interesting antiviral activity that could be exploited as possible drug candidates for COVID-19 treatment.

Coronaviruses (CoVs) are enveloped viruses belonging to the Nidovirales order and are divided into four genera based on phylogeny (https://talk.ictvonline.org/p/coronavirus-genomes). CoVs have been detected in a wide spectrum of mammals and avian species such as dogs, cats, pigs, chickens, cows, camels, bats, minks and/or pangolins, and cause severe diseases including gastroenteritis and respiratory tract diseases. Seven human coronaviruses (HCoVs) have been identified to date (HCoV-NL63, HCoV-229E, HCoV-HKU1, HCoV-OC43, Middle East respiratory syndrome coronavirus (MERS-CoV) and severe acute respiratory syndrome coronaviruses 1 and 2 (SARS-CoV-1 and SARS-CoV-2)). Their virions are about 120–160 nm in diameter and contain a linear, positive, single-stranded RNA genome of ≈26–32 kilobases, which encodes 16 non-structural proteins (nsp1 to nsp16), and four or five structural proteins including the spike (S), envelope (E), membrane (M), nucleocapsid (N) and, for HCoV-OC43 and HCoV-HKU1, the hemagglutinin (HE) [1–4].

Currently, there are no specific antiviral drugs that target HCoV viruses. However, large *in silico* screening studies and numerous *in vitro* studies have assessed the antiviral activity of various drugs on HCoV. These studies have accelerated substantially since the beginning of the COVID-19 pandemic at the end of 2019. Drug repurposing represents the most pertinent strategy, due to the reduced time for approval by international medicines agencies, the low cost of development, and low risk, in comparison with the *de novo* design of new molecules for which clinical trials to test efficacy and safety are not possible within the context of a health emergency. Drug repurposing offers an opportunity to by-pass pre-clinical tests, by using molecules whose toxicity is already well known.

## Materials & methods

### Bibliographic searches

On 25 July 2021, searches were carried out on PubMed and Google Scholar using the combined keywords coronaviruses, human coronaviruses, severe acute respiratory syndrome coronavirus, SARS-CoV, SARS-CoV-2, *in vitro*, cell culture, sensitivity assay, drug repurposing, drug repositioning, *in silico* and computational. The results of these searches were completed with data collected from the Stanford coronavirus antiviral research database on 25 July 2021 (https://covdb.stanford.edu). The sensitivity assays carried out on interferons and monoclonal antibodies were excluded. We reviewed the following: 1) the molecules tested in at least four sensitivity assays (Table 1). 2) The most potent molecules based on effective concentration 50 (EC_50_), which is the compound concentration that is required to inhibit viral RNA replication by 50% (Table 2).

**Table 1.** List of the compounds tested at least four times in sensitivity assays on cell cultures for SARS-CoV-1 and SARS-CoV-2.

Compound	SARS-CoV-1	SARS-CoV-2	Status
Amlodipine		6	Approved
Amodiaquine		7	Approved, investigational
Artesunate		8	Approved, investigational
Atazanavir		4	Approved
Chloroquine		33	Approved, investigational, vet approved
Chlorpromazine		4	Approved, investigational, vet approved
Ciclesonide		4	Approved, investigational
Clofazimine		6	Approved, investigational
Cyclosporin		4	Approved, investigational, vet approved
Ebastine		4	Approved, investigational
Favipiravir		11	Approved, investigational
Hydroxychloroquine		21	Approved
Loperamide		6	Approved
Lopinavir		12	Approved
Mefloquine		5	Approved, investigational
Nelfinavir		5	Approved
Niclosamide		4	Approved, investigational, vet approved
Remdesivir	1	51	Approved investigational
Ribavirin		6	Approved
Ritonavir		4	Approved, investigational
Sofosbuvir		4	Approved
Tafenoquine		4	Approved, investigational
Trifluoperazine		4	Approved, investigational
Alpha-1 antitrypsin		4	Investigational
Apilimod		8	Investigational
AZD8055		4	Investigational
Pyronaridine		5	Investigational
Nafamostat	1	11	Investigational
Beta-D-N4-Hydroxycytidine	1	6	Experimental
Camostat	1	7	Experimental
GS-441524		11	Experimental
Aprotinin	1	7	Approved, investigational then withdrawn
Terfenadine		4	Approved then withdrawn
Berbamine		4	Not listed
Cepharanthine		7	not listed
GC376		7	Not listed
K11777	2	7	Not listed
RS-504393		4	Not listed
VBY-825		4	Not listed
Z-FA-FMK		5	Not listed
Salinomycin		4	Vet approved

The status mentioned in the third column was based on the DrugBank site (https://go.drugbank.com/drugs).

**Table 2.** Most potent compounds on SARS-CoV-2 according to *in vitro* sensitivity assays that obtained EC_50_ <3 μm, and corresponding computational predictions.

Compound	Status	Sensitivity assays				Predicted by molecular docking
		Minimal EC_50_ (μM)	Cells	Maximal EC_50_ (μM)	Cells	
**Anthelminthic**						
Niclosamide	Approved, investigational, vet Approved	0.09	Vero E6	0.3	Vero	No
Ivermectin	Approved	1.7	Vero E6	2	Vero/hSLAM	Yes – Kadioglu (https://www.who.int/bulletin/online_first/20-255943.pdf)
**Antiarrythmics**						
Amiodarone	Approved, investigational	0.05	Huh7	>100	Vero	No
Verapamil	Approved	0.5	Huh7			No
**Antibacterial**						
Nigericin	Experimental	0.09	Vero E6/TMPRSS2			No
Brilacidin	Investigational	0.6	Calu-3	∼5	Vero	No
Azithromycin	Approved	2.1	Vero E6			Yes – El-hoshoudy, *J. Mol. Liquids* (2020); Fantini, *Int. J. Antimicrob. Agents* (2020); Bezerra Braz, *Int. J. Antimicrob. Agents* (2020)
Clofazimine	Approved, investigational	0.01	Vero	<5	Cardiomyocytes	Yes – Hosseini, Life Science (2020)
Lasalocid	Vet approved	0.4	Vero E6/TMPRSS2			No
Salinomycin	Vet approved	0.003	Calu-3	0.2	Vero CCL81	No
Monensin	Experimental, vet approved	0.1	Vero E6/TMPRSS2			No
Monensin sodium salt	Experimental, vet approved	0.6	Vero			No
Narasin	Experimental, vet approved	0.07	Vero E6/TMPRSS2			No
Indanomycin	Not listed	0.6	Vero E6/TMPRSS2			No
**Antidepressant**						
Clomipramine	Approved	2	A549/ACE2	14	Vero E6	No
Trimipramine	Approved	1.5	A549/ACE2	10	Vero E6	No
Indatraline	Not listed	1.6	Vero E6/TMPRSS2			No
**Antifibrinolytic**						
Aprotinin	Approved, investigational, withdrawn	0.5	Caco-2			No
Aprotinin/Omp	Approved, investigational, withdrawn	0.2	Caco-2			No
α-1 antitrypsin	Investigational	0.8	Vero E6	>20	Caco-2	No
Nafamostat	Investigational	0.002	Calu-3	>100	Vero E6/TMPRSS2	No
Camostat	Experimental	0.3	Calu-3	>50	Vero	No
**Antifungal**						
Ketoconazole	Approved, investigational	2.4	Caco-2			No
Cycloheximide	Not listed	0.6	Caco-2			No
**Antihistaminic**						
Desloratadine	Approved, Investigational	0.9	A549/ACE2			No
Ebastine	Approved, investigational	1.2	Huh7.5	6.9	Vero	No
Astemizole	Withdrawn	0.9	293T/ACE2	∼1.2	Vero E6	No
Azelastine	Approved	2.4	A549/ACE2			No
**Antiinflammatory**						
Celecoxib	Approved	0.04	Vero			Yes – Gimeno, *Int. J. Mol. Sci.* (2020)
Auranofin	Approved, investigational	1.4	Huh7			No
**Antineoplastic**						
Brequinar	Experimental	0.3	Vero E6			No
Gemcitabine	Approved	1.2	Vero E6			No
Thioguanine	Approved	0.2	Huh7			No
Tamoxifen citrate	Approved	1.8	Vero E6	34	Vero E6	No
Bemcentinib	Investigational	0.1	Huh7.5	>50	Vero E6	No
Naquotinib	Investigational	0.06	Huh7.5			No
Tamibarotene	Investigational	2.5	Vero E6			
Tretinoin	Approved, investigational, nutraceutical	1	Vero E6			Yes – Dey, *Comput. Biol. Med.* (2020)
Raloxifene HCl	Approved, investigational	0.02	Vero			No
Bafetinib	Investigational	2.2	A549/ACE2			No
Bosutinib	Approved	0.02	Huh7			No
Fedratinib	Approved, investigational	0.02	Huh7			No
Gilteritinib	Approved, investigational	0.2	Huh7	>50	Calu-3	No
Dacomitinib	Approved, investigational	0.04	Calu-3	0.8	Huh7.5	No
Lapatinib	Approved, investigational	1.6	A549/ACE2			No
Nilotinib	Approved, investigational	0.08	Vero E6			Yes – Ruan, *J. Med. Virol.* (2020); Wei, *Chin. J. Integr. Med.* (2020)
Abiraterone acetate	Approved	1.9	Vero E6	7.1	Vero E6	No
Berzosertib	Investigational	0.005	Vero E6	0.7	Vero E6	No
Temsirolimus	Approved	2.9	Vero			No
Vistusertib	Investigational	0.02	Vero E6			No
**Antiparkinson agent**						
Benztropine	Approved	1.8	A549/ACE2			No
**Antiprotozoal**						
Nitazoxanide	Approved	1	Vero E6	4.9	Vero E6	No
Emetine	Experimental; not approved	0.5	Vero E6			Yes – Das, *Journal of Biomolecular Structure and Dynamics* (2020)
Suramin	Investigational	2.9	Vero E6	20	Vero E6	No
Diiodohydroxyquinoline	Approved	1.4	Vero E6			No
Pyronaridine	Investigational	0.2	Huh7.5	8.6	Calu-3	Yes – Hosseini, *Life Sciences* (2020)
Piperaquine (in combination with dihydroartemisinin)	Experimental, investigational -	2.1	Huh7.5			No
Maduramycin	Vet approved	0.06	Vero E6/TMPRSS2			No
Amodiaquine	Approved, investigational	0.6	Huh7.5	>50	Calu-3	Yes – Peele, *Inform. Med. Unlocked.* (2020)
Hydroxychloroquine	Approved	0.2	Huh7.5	<10	Vero E6	El-hoshoudy, *J. Mol. Liquids* (2020); Bezerra Braz, *Int. J. Antimicrob. Agents* (2020); Chitranshi, *J. Transl. Med.* (2020); Fantini, *Int. J. Antimicrob. Agents* (2020)
Tafenoquine	Approved, investigational	2.5	Vero E6	16	Vero E6	No
Chloroquine	Approved, investigational, vet approved	0.1	Vero E6	>50	Vero E6	Yes – Li *et al.* (preprint) (2020); El-hoshoudy, *J. Mol. Liquids* (2020); Bezerra Braz, *Int. J. Antimicrob. Agents* (2020); Chitranshi, *J. Transl. Med.* (2020)
Artesunate	Approved, investigational	0.5	Calu-3	53	Vero	No
Halofantrine	Approved	0.3	HeLa-ACE2			No
**Antipsychotic**						
Flupenthixol	Approved, investigational, withdrawn in the USA	0.6	A549/ACE2			No
Thioridazine HCl	Withdrawn	2.2	Vero			No
Elopiprazole	Not listed	1.6	Vero E6	2.7	Huh-7/ACE2	No
Metoclopramide	Approved investigational	0.5	Huh7			No
**Antiseptic (topical)**						
Hexachlorophene	Withdrawn	0.9	Vero	1.5	Calu-3	No
**Antiviral**						
Atazanavir/r	Approved	0.5	Vero E6	0,6	A549	No
Daclatasvir	Approved	0.6	Huh7	1.1	Calu-3	No
Remdesivir	Approved, investigational	0.002	Huh7.5	<20	HAE	Yes – Hall, *Travel Medicine and Infectious Disease* (2020); El-hoshoudy, *J. Mol. Liquids* (2020); Chitranshi, *J. Transl. Med.* (2020); Elfiky, *Life Sciences* (2020)
Diltiazem + remdesivir	Approved, investigational	0.3	Vero	0.7	Vero	No
Remdesivir/Omp	Approved, investigational	0.02	Caco-2			No
Entecavir	Approved, investigational	0.04	Huh7	>20	Vero	No
Boceprevir	Approved, withdrawn	1.9	Vero 76			Yes – Eleftheriou, *Molecules* (2020); Elfiky *et al.* (2020).
Lopinavir	Approved	1.7	Vero E6/TMPRSS2	>50	Vero E6	Das, *Journal of Biomolecular Structure and Dynamics* (2020); Hakmi, *Bioinformation* (2020); El-hoshoudy, *J. Mol. Liquids* (2020); Eleftheriou, *Molecules* (2020); Chitranshi, *J. Transl. Med.* (2020); Peele, *Inform. Med. Unlocked* (2020)
Atazanavir	Approved	0.2	A549	>50	Vero	No
Nelfinavir	Approved	0.8	Vero E6/TMPRSS2	>50	Vero E6	Yes – Musarrat, *J. Med. Virol.* (2020); Huynh, *J. Phys. Chem. Lett.* (2020)
**Cardiac glycoside**						
Ouabain octahydrate	Ouabain approved	0.02	Vero			No
Ouabain	Approved	0.02	Vero	<0.1	Vero	No
Digoxin	Approved-cardiac glycoside	0.04	Vero	0.2	Vero	No
Digitoxin	Approved, investigational	0.1	Vero	0.2	Vero	Yes – Wei, *Chin. J. Integr. Med.* (2020)
**Hemostatic**						
Polidocanol	Approved	0.2	Caco-2			No
**Hypolipidemic agent**						
Lomitapide	Approved, investigational	0.8	Huh7			No
**Immunosuppressant**						
Cyclosporin	Approved, investigational, vet approved	0.2	Calu-3	5.8	Vero	Yes – El-hoshoudy, *J. Mol. Liquids* (2020)
Mycophenolate	Approved	0.9	Vero E6/TMPRSS2			Yes, Elfiky *et al.* (2020)
**Interleukin inhibitor**						
Apilimod	Investigational	0.007	A549/ACE2	∼0.01	Vero E6	No
**Pancreatic lipase inhibitor**						
Cetilistat	Investigational	1.1	Vero E6			No
**Antispasmodic**						
Ethaverine	Approved (France, Germany, Spain)	0.6	Caco-2			No
**Others**						
Hanfangchin A (Tetrandrine)	Experimental	0.6	Huh-7/ACE2	1.2	Vero E6	No
Almitrine	Approved	1.4	Caco-2			No
Acitretin	Approved	2.5	Vero E6			
Pristimerin	Not listed	0.1	Vero E6			No
Lycorine	Not listed	0.3	Vero E6			No
Cepharanthine	Not listed	0.01	Huh7.5	30	Calu-3	Yes – Ruan, *J. Med. Virol.* (2020)
Homorringtonine	Approved, investigational	2.1	Vero E6	2.5	Vero E6	No
Leupeptin Hemisulfate	Not listed	0.03	Huh7.5			No
Nanchangmycin	Not listed	0.01	Vero CCL81	0.07	Vero E6/TMPRSS2	No
Lactoferrin	Not listed	0.3	Huh7			No
Griffithsin	Not listed	0.06	Vero E6			No
Liquiritin	Not listed	2.4	Vero E6			No

The main data provided on pharmacodynamics and drug toxicity were collected from the DrugBank database (https://go.drugbank.com/drugs/DB00836) [1]. Finally, we added results from the first clinical studies available on 25 July 2021.

### Analysis methods used in *in vitro* sensitivity assays performed on cell cultures

Several essential parameters used for *in vitro* test sensitivity assays varied according to studies. These parameters include cell lines, the multiplicity of infection (MOI: infectious virus titer divided by the number of cells), the time between addition of the drug and incubation until addition of viruses, drug concentration, endpoint for evaluation of viral replication and the read-out system for the assessment of viral replication.

The cells used in each *in vitro* sensitivity assay have to be adapted according to the virus tested: the condition to be used is that cells have to be permissive for the virus tested, implying that they harbor the virus cell receptor specific for the virus tested.

**Figure 1.**
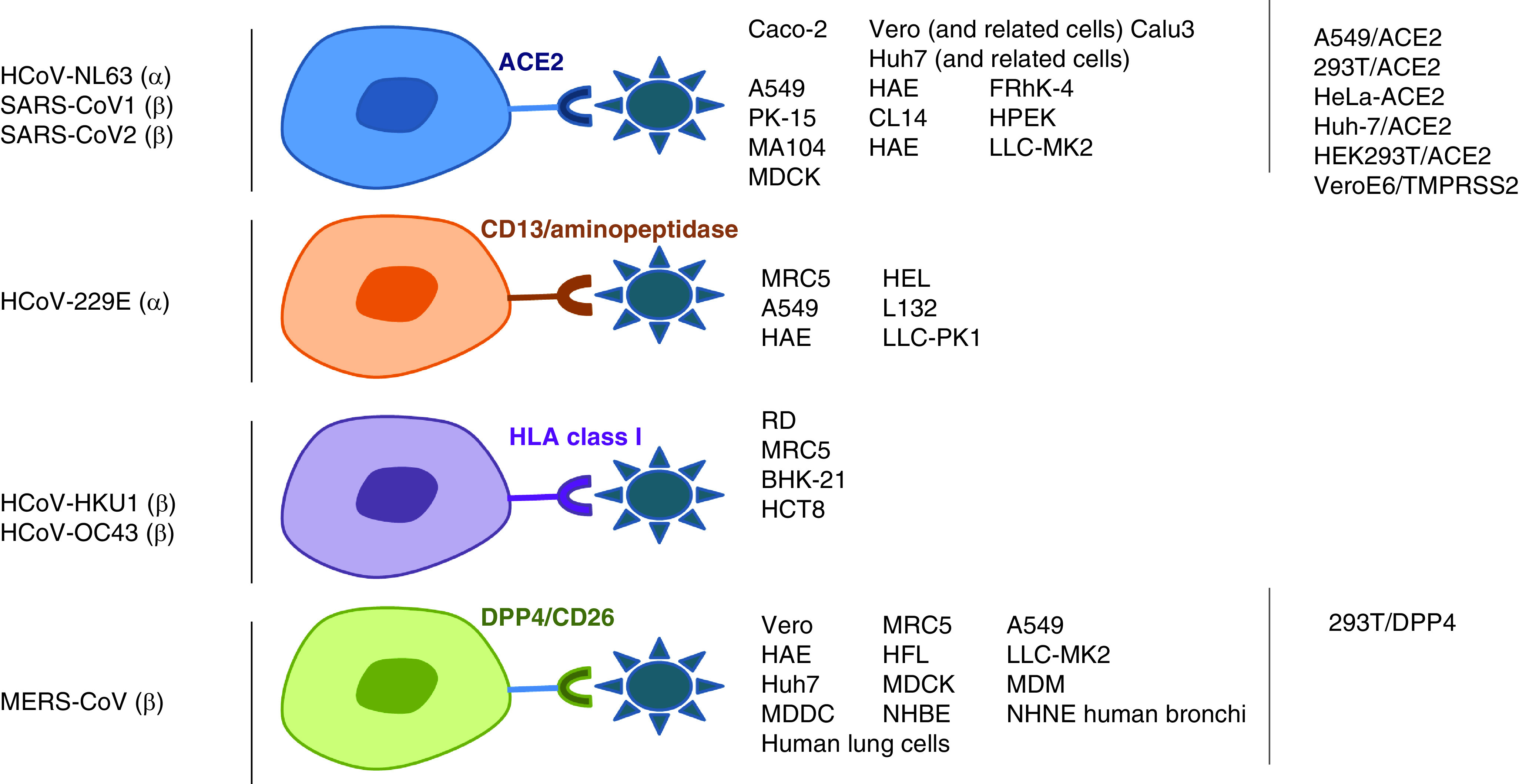
Cell lines that can be used in sensitivity assays for human coronaviruses, according to their cell receptors.

**Figure 2.**
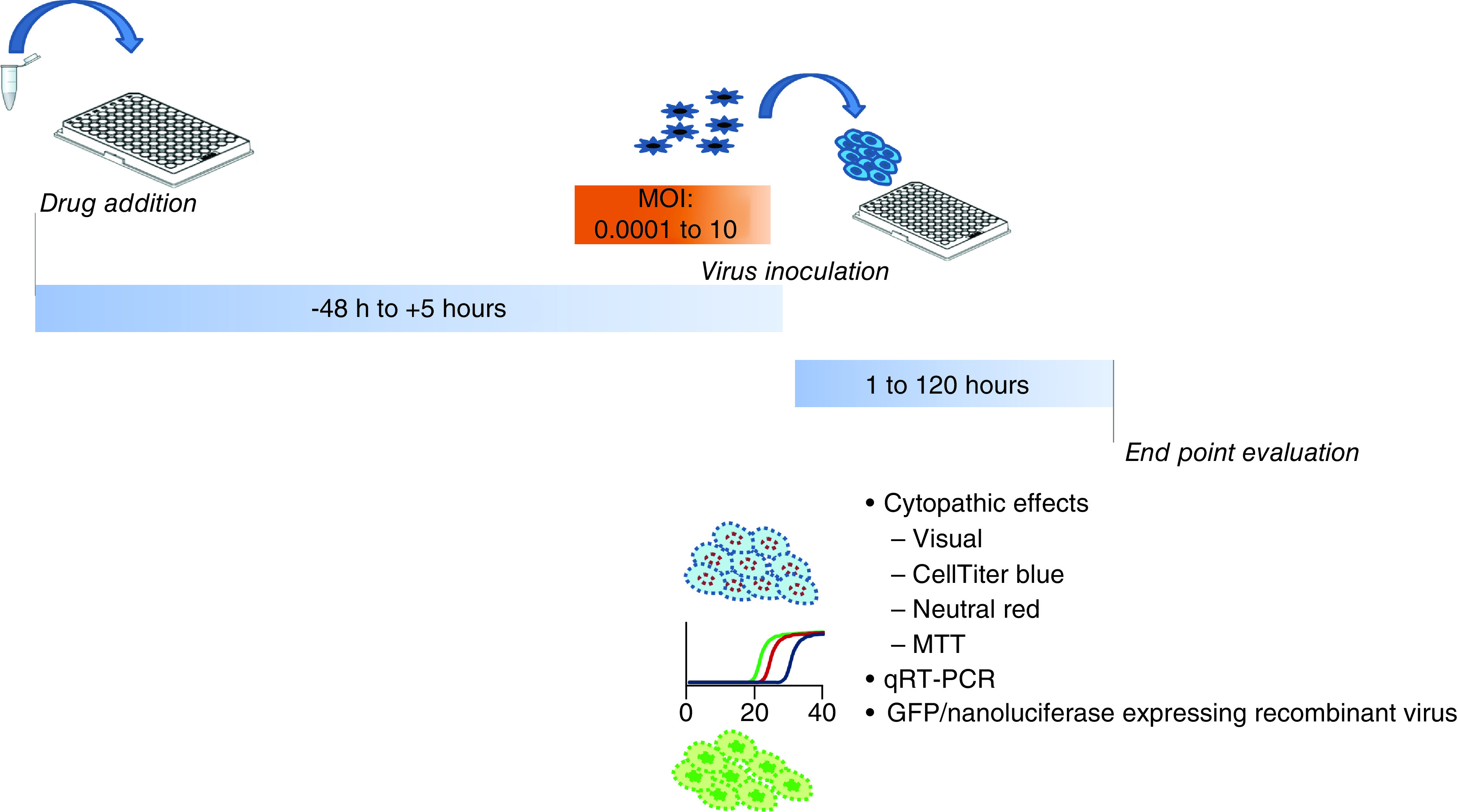
Representation of the discrepant parameters used in sensitivity assays.

Most of the studies that assessed the antiviral activity of molecules against HCoVs used Vero E6 cells (kidney epithelial cells from African green monkeys), a cell line largely used to propagate many viruses, mainly due to their IFN-deficiency, and thus have a high permissivity to MERS-CoV, SARS-CoV-1 and SARS-CoV-2. For SARS-CoV-2, a large panel of other cells were used, either naturally expressing the cellular receptor for the virus, or transfected with the human *ACE2* gene. All the cell lines used in the sensitivity assays are summarized in Figure 1. Primary cell lines have been little used for coronavirus sensitivity assays. Primary cell culture is the *ex vivo* culture of cells derived from tissue explants. The human airway epithelial (HAE) primary cell line is a pseudostratified mucociliary epithelium that was used for SARS-CoV-1 and SARS-CoV-2 [2–4]. In addition, human embryonic stem cell-derived cardiomyocytes and *ex vivo* lung cultures have also been used for sensitivity assays [5]. All the discrepancies on the other parameters are summarized in Figure 2.

The multiplicity of infection (MOI) of the virus was not standardized and varied greatly depending on the virus and the study, ranging from 0.0001 to 10. The MOI mostly used for SARS-CoV-1 and SARS-CoV-2 was 0.01. It should be noted that some studies do not mention the MOI used [6].

The time of drug incubation was also a variable parameter (from 48 h before to 5 h after virus inoculation). However, most of the studies used a 1 h incubation of cells with the compounds before virus inoculation. In most assays, dose response curves were performed with the aim of calculating the EC_50_, which requires the assessment of compound dilutions. Most studies also evaluate the CC_50_, which is the concentration that reduces the total cell number by 50%. However, for some experiments that evaluated only one compound concentration, the EC_50_ could not be calculated, although the percentage of virus inhibition with the compound concentration used is generally reported.

Viral replication could be assessed through an evaluation of cytopathic effects with CellTiter-Blue (PROMEGA), neutral red, MTT (4,5-dimethylthiazol-2-yl)-2,5-diphenyltetrazolium) or visually. CellTiter-Blue is a commercial fluorescent assay to monitor cell viability. Neutral red is taken up by viable cells, the ability to incorporate neutral red decreasing as lysis occurs. A colorimetric quantitation will be inversely correlated to the cytopathic effects. MTT enters cells, is reduced by NAD(P)H-dependent oxidoreductase in formazan and turns purple; this coloration will be quantified by a spectrophotometric evaluation. Importantly, it should be noted that the sensitivity thresholds of the detection of cytopathic effects could be different with a visual inspection rather than a fluorescence detection performed with the CellTiterGlo. This difference according to the method of detection had already been reported [7].

Other studies assessed infectious viruses by determining the viral titer at the end of the experiment. Viral replication could also be assessed by virus expression: either by RNA quantification, or the expression of a viral protein. RNA quantification performed by quantitative RT-PCR allows to compare the viral load in control cells infected by the virus with those in the presence of the tested compound. The differences between the viral loads allows to assess the percentage of inhibition of the viral replication. Some studies used green fluorescent protein (GFP) or nanoluciferase-expressing recombinant viral strains, the viral expression being quantified by fluorometry at the end-point evaluation. Finally, a few teams still use plaque reduction assays.

Another parameter should also be considered. The viral strain was not the same in all the studies, despite the fact that these viruses accumulate mutations and that many variants exist. Only very few studies were found to test several strains [5].

### Molecular docking

Interestingly, these approaches can supplement *in vitro* cell sensitivity assays by predicting the interactions of drugs in pathways important in the viral replication cycle. They are complementary and have the added value of targeting the most interesting repositioned drug candidates. Targeted candidates can subsequently be tested by sensitivity assays on cell cultures.

The study of protein–protein interactions could be useful in terms of targeting possible therapeutic options. The objective is to clone, tag and express SARS-CoV-2 proteins in human cells to identify human proteins that are physically associated with them, using affinity-purification mass spectrometry. On the high-confidence protein–protein interactions that are identified, sensitivity assays are then carried out on cell cultures that were used to confirm these results [8].

The strategy could be purely based on digital predictions. Computational tools have also been used to better understand the interaction between drugs and viral or host proteins. Molecular docking and molecular dynamic simulations have been used to find binding energies resulting from the simulated interactions of several thousands of compounds, with the viral or host proteins [9].

Other studies investigated whether SARS-CoV-2 proteins could interact with targeted pathways previously shown to be essential for CoVs replication cycles, which were the unfolded protein response (UPR), the mitochondrial permeability transition pores (MPTP), the NLRP-3 inflammasome and autophagy of the host cells. After that, the objective was to identify drugs known to modulate these pathways [10]. The most widely used strategy was to screen a database of several hundreds or thousands of compounds and to predict binding affinity with the viral or host proteins involved in the viral replication cycle.

## Results

**Figure 3.**
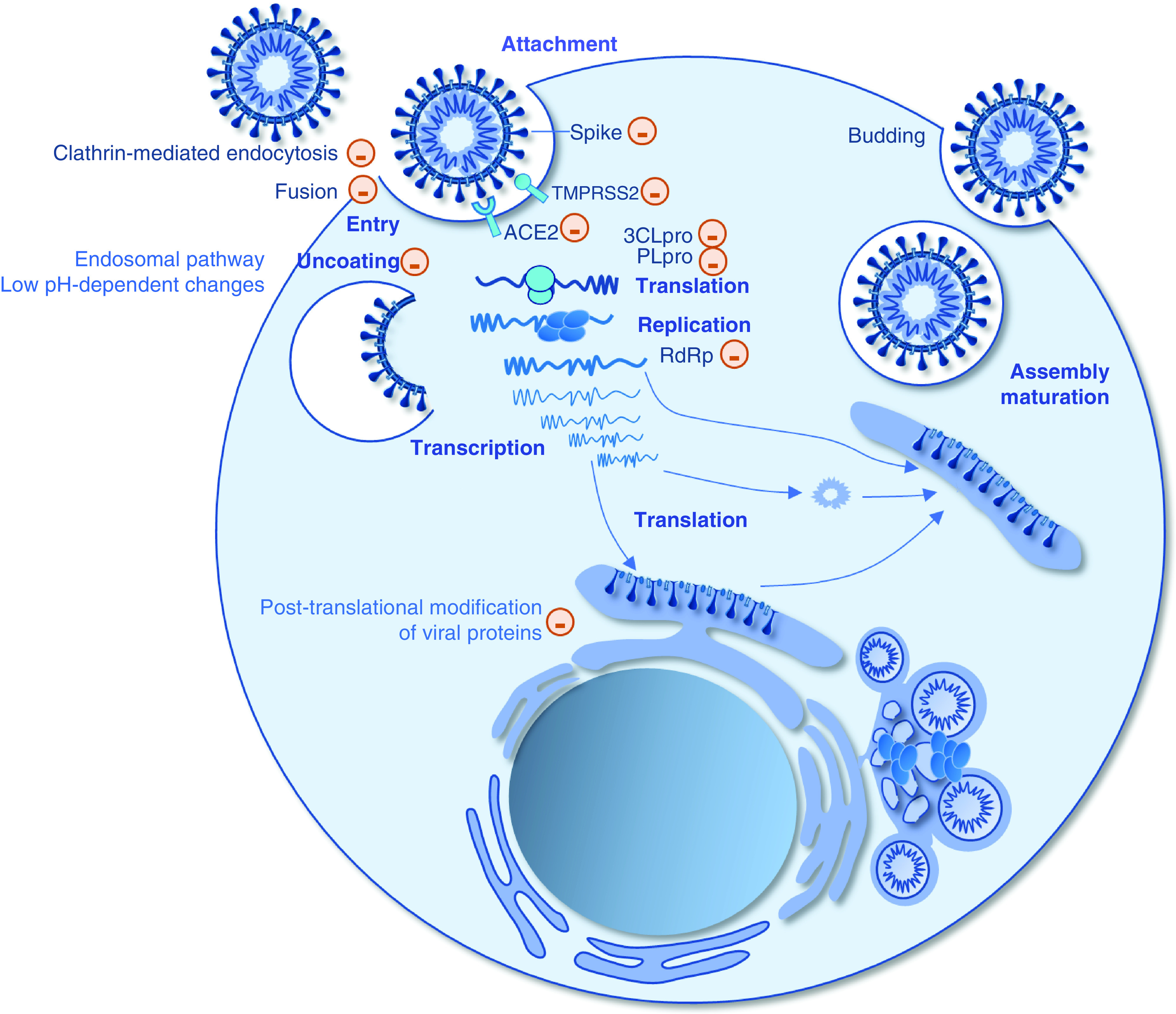
Schematic of the replicative cycle of SARS-CoV-2 and evidenced or putative sites of action of drugs.

Although a few anti-HCoV drugs can act directly on the viral proteins, most drugs interfere with the cell metabolism and the molecular crosstalk taking place between the virus and the target cell. Alongside sensitivity assays in cell culture, dozens of molecular docking studies have been carried out. Among the viral proteins involved in molecular docking studies, the 3-chymotrypsin-like protease (3CL-Pro), the papaïn-like protease (PL-Pro), the RNA-dependent RNA polymerase (RdRp) and the spike protein have been deeply explored as druggable targets (Figure 3). To our knowledge, 228 main compounds have been found by computational studies to be drug candidates (Supplementary Table 1).

### Antivirals

#### RdRP inhibitors

##### Remdesivir

Remdesivir (GS-5734) is a parenteral phosphate prodrug of an adenosine analog acting as a chain terminator of the RNA polymerase, at position i + 3. The 1′-cyano group of remdesivir sterically clashes with the complex RdRp, inhibiting further enzyme translocation and terminating replication. Remdesivir was initially described as a potential treatment for Ebola [11–13]. It displays *in vitro* activity against several viral families including Arenaviridae, Flaviviridae, Filoviridae, Paramyxoviridae, Pneumoviridae and Coronaviridae. Its anticoronal activity was largely tested in cell cultures with, to date, four sensitivity assays performed on SARS-CoV-1 [14–16] and MERS-CoV [14,17,18], and 49 on SARS-CoV-2 [3,16,19–45]. Remdesivir inhibits *in vitro* the replication of the MERS-CoV on Calu3 and HAE cells with EC_50_ comprised between 0.03 and 0.09 μM with high SI.

The EC_50_ varied from 0.002 μM when tested with a MOI = 1 on Huh7.5 cells and an evaluation by a visual inspection of the cytopathic effects [29] and 27 μM when tested with a MOI = 0.02 on Vero E6 cells and an evaluation based on virus performed by RNA expression quantification [19]. Two studies were carried out on Human Airway Epithelial cells [3,46]. One of them using a MOI of 0.5 provided an EC_50_ of 0.01 μM and a selectivity index >1000 [24].

After a single 2-h intravenous infusion of 75 mg of remdesivir, the plasmatic concentration of the prodrug remdesivir peaks at 2.5 μM (2,229 ng/ml), which is expected to achieve an antiviral efficacy according to the EC_50_ obtained *in vitro* [47].

Combinations using remdesivir were also found to have antiviral efficacy. Choy *et al.* found a synergy between remdesivir at 6.25 μM and emetine at 0.195 μM with 64.9% inhibition in viral yield [19]. In addition, one study reported that the combination of remdesivir and diltiazem revealed a synergistic antiviral activity against SARS-CoV-2 across a wide dose range that remained below remdesivir therapeutic plasma concentrations [48,49].

Diltiazem is a Ca^2+^ channel blocker commonly used in the treatment of hypertension for which antiviral activity was recently demonstrated against the Influenza virus in A549 human lung epithelial cells with a very low EC_50_ of 0.84 nM and which was confirmed in primary cells of reconstituted Human Airway Epithelia [50]. Its antiviral properties may be based on the capacity to induce type I and III interferon antiviral responses [50].

Remdesivir has been predicted to bind to the RNA-dependent RNA polymerase of SARS-CoV-2 with a binding energy of -7.6 kcal/mol, which possibly may inhibit its function [51], but also with the main viral protease with a binding energy ranging from -6.4 to -7.2 kcal/mol [52–54].

After a clinical trial that had shown the superiority of remdesivir compared with placebo [55,56], the SOLIDARITY clinical trial reached to the conclusion that remdesivir had little or no effect for hospitalized patients, when considering time for discharge, initiation of ventilation and mortality [57].

##### Favipiravir

Favipiravir is a direct acting antiviral with the structure of a modified pyrazine analog. This prodrug requires activation by ribosylation and phosphorylation to provide the active favipiravir-RTP. The active metabolite acts as a nucleotide analog, binds and inhibits the RNA-dependent RNA polymerase, thus preventing viral replication, through a combination of chain termination and lethal mutagenesis by causing C-to-U and G-to-A transitions in the SARS-CoV-2 genome. It was also suggested as acting as an entry inhibitor for SARS-CoV-2. It was initially developed to treat Influenza infections. Maximal plasmatic concentration reaches 51.5 μg/ml.

Given that the catalytic domain RdRp of Influenza virus may be similar to those of other RNA viruses, favipiravir has been investigated for the treatment of infections with Ebola virus, Lassa virus and SARS-CoV-2. Although it is currently being investigated through clinical trials, *in vitro* sensitivity assays have revealed contradictory results. An antiviral effect was concluded by Driouich *et al.* with a strong dose effect. The sensitivity assay on Vero E6 cells was based on a reduction of the cytopathic effect, with a MOI of 0.001, an EC_50_ was 32 μg/ml, and with a MOI of 0.01, it was 70 μg/ml (CC_50_ >78.5 μg/ml) [58]. Shannon *et al.* also drew conclusions as to the efficacy of favipiravir, despite the high EC_50_ of 207 μM when the evaluation was based on the visualisation of cytopathic effects and 118 μM when it was based on an evaluation of RNA quantification [59].

With similar results, Wang *et al.* were more cautious, concluding that the reduction of viral infection in Vero E6 at a MOI of 0.05 was shown only with high concentrations (EC_50_ = 61.88 μM, CC_50_ >400 μM, SI >6.46). Efficacy was evaluated by viral quantification in the cell supernatant by qRT-PCR and confirmed by virus nucleoprotein expression visualized through immunofluorescence microscopy [21].

In contrast, Choy *et al.* showed that favipiravir at a high concentration (100 μM) had no antiviral efficacy against SARS-CoV-2 when MOI = 0.02 on the same cell lines (Vero E6), with an evaluation by both quantitation of infectious virus and RNA quantification [19]. Zandi *et al.*, Jeon *et al.*, and Liu and Ohashi also concluded that there was no antiviral efficacy [20,38,60,61]. A meta-analysis, which included nine clinical studies, showed a benefit in patients treated with favipiravir especially on viral clearance, oxygen requirement, and in the mortality that was approximately 30% less than in the control group, but these differences were not significant and probably due to a delayed use of the molecule. However, favipiravir treatment was interestingly associated with a significant clinical improvement during 7 days after hospitalization [62].

##### Ribavirin

Ribavirin is a guanosine analog that needs to be activated by an adenosine kinase, and that blocks viral RNA synthesis and viral mRNA capping. It displays a broad antiviral spectrum against several RNA and DNA viruses. It was initially indicated in the treatment of HCV infections. It is currently used as an adjunct therapy to several new direct acting antivirals targeting HCV. The main toxicity is the risk of anemia.

Ribavirin clearly does not inhibit SARS-CoV-1 replication in Vero, Vero E6 or Vero 76 cells at high concentrations, with an EC_50_ ranging from 82 μM to >4095 μM [63–68]. However, one study concluded that ribavirin could inhibit SARS-CoV-1 replication in other cells (PK15, Caco-2, CL14 and HPEK), with EC_50_s ranging from 2.2 to 9.4 μg/ml at a MOI = 0.01, and that high concentrations of ribavirin (50 μg/ml) completely suppressed the formation of cytopathic effects in all cell lines infected with two strains of SARS-CoV-1 [66].

For SARS-CoV-2, although some clinical trials are currently evaluating the efficacy of ribavirin against COVID-19, no antiviral activity of ribavirin against SARS-CoV-2 has been clearly demonstrated. Pizzorno *et al.* concluded that there was an absence of antiviral efficacy of this compound with an EC_50_ >10 μM tested on Vero E6 cells with a MOI of 0.01 [3]. Similarly, Choy *et al.* did not find any SARS-CoV-2 inhibition at 100 μM [19]. Wang observed that only a high concentration of ribavirin was required to reduce SARS-CoV-2 infection (EC_50_ = 109.50 μM, CC50 >400 μM, SI >3.65) [21]. Congruently, Zandi *et al.* found that 20 μM of ribavirin showed only 12% inhibitory effect on the SARS-CoV-2 with concomitant cytotoxicity [61].

##### Sofosbuvir

Although, sofosbuvir was shown *in silico*, to tightly bind to SARS-CoV-2 RdRp with a binding energy of -7.5 kcal/mol, the antihepatitis C virus nucleotide analog does not harbor any inhibitory activity in cell culture with EC_50_ comprised between 6.2 μM [69] on Huh7 and >200 μM on Vero E6 cells [38]. Moreover, the combinations currently used for hepatitis C treatment of sofosbuvir + velpatasvir and sofosbuvir + ledipasvir, did not show any activity against SARS-CoV-2 with EC_50_ >50 μM on Vero E6 cells [38].

A clinical trial assessing the combination sofosbuvir + daclatasvir shortened the median duration of hospitalization in a group of 33 hospitalized adults with moderate to severe disease who were not receiving mechanical ventilation, compared to the control group that was receiving the standard of care [70]. Another study showed a trend in fewer hospitalizations in the sofosbuvir/daclatasvir arm, but without any significant difference [71].

##### Entecavir

Entecavir is a nucleoside analog used for the treatment of Hepatitis B viral hepatitis. The EC_50_ of entecavir against SARS-CoV-2 on Huh7 cells was 0.04 μM at MOI 0.2 with an evaluation by viral expression (N protein) [25].

###### Viral protease inhibitors

In host cells, the released viral polyproteins are cleaved into individual active nsps. CoVs possess two proteases: PLpro (Papaïn-like serine protease or nsp3), and chymotrypsin-like cysteine protease (3CLpro, Main MPro or nsp5) that play an essential role in the cleavage of polyproteins. Several protease inhibitors have been identified *in silico* and *in vitro* as potential therapeutics for MERS-CoV, SARS-CoV-1 and SARS-CoV-2. The conserved cysteine residues of the PLPro are useful for binding the Zn2+ ion (structural role) and for catalytic role. PLPro inhibitors can act by ejecting Zn2+ ion from the Zn site or by blocking of the Cys residue.

### Lopinavir

Lopinavir was with remdesivir, among the first antiviral tested on SARS-CoV-2. It is an inhibitor of the HIV protease. It is marketed in association with another protease inhibitor, ritonavir, which acts as an enzymatic inhibitor, thus boosting the plasmatic concentration of lopinavir. Molecular docking demonstrated that ritonavir may also bind to the SARS-CoV-2 PLPro with a docking-free energy of -6.9 kcal.mol^-1^, thus inhibiting viral replication [72]. Lopinavir showed antiviral activity against SARS-CoV-1 *in vitro*, with EC_50_ varying from 1.7 on Vero E6/TMPRSS2 at MOI 0.01 [60] to >50 μM on Vero E6 and does not mention the MOI [73], moreover with a low specificity index (<0.1). Choy *et al.* observed antiviral activity of lopinavir against SARS-CoV-2 *in vitro* with an EC_50_ = 26 μM on Vero E6 cells [19], but with a low selectivity index. The antiviral effect could therefore be due to cytotoxicity. Moreover, the EC_50_ could not be expected to be lower than the plasmatic concentration obtained with 400/100 mg of lopinavir/ritonavir dosing and thus may not achieve the antiviral activity *in vivo*. It was virtually predicted that lopinavir would exhibit a low free-binding energy to the active site of the SARS-CoV-2 main protease, ranging from -7.57 to -9.9 kcal/mol, by binding near the crucial catalytic residues, HIS41 and CYS145 [53,74–77], but also with Thr26, Gly143 and Ser144 [54]. Moreover, it also could act as an entry inhibitor for SARS-CoV-2. Congruently, the combination lopinavir/ritonavir did not demonstrate any benefit in hospitalized adults with severe Covid-19 [78], neither in mortality, duration of hospital stay, or progression to invasive mechanical ventilation [79].

#### Atazanavir

Atazanavir, an inhibitor of the HIV protease, exhibits a EC_50_ = 0.2 μM against SARS-CoV-2 at MOI = 0.01 on A549 cells, evaluated by virus expression. In addition, antiviral activity was reduced when using the combination of atazanavir and ritonavir with an EC_50_ = 0.6 μM in the same experimental conditions [80]. The EC_50_ was considerably increased on Vero E6/TMPRSS2 with an EC_50_ of 9.4 μM at an MOI of 0.01 [81]. To note, the EC_50_ on Vero cells (>50 μM) was more than 5–50-fold higher than those in other cells [20,80,81].

#### Nelfinavir

Nelfinavir is a protease inhibitor effective against HIV. It could also act against the SARS-CoV-1 3CL protease. With an MOI of 0.01, nelfinavir has an activity against SARS-CoV-1 with low EC_50_ of 0.05 and 1.1 on Vero E6 and Vero E6/TMPRSS2, respectively [82]. Its activity has also been shown against SARS-CoV-2 on the same cell lines and on HeLa-ACE2 [26,34,38,60,82]. Docking studies have predicted that nelfinavir may inhibit the main protease with a binding energy of -7 kcal/mol [83], but also unexpectedly binds near the SARS-CoV-2 spike protein [84].

#### Other protease inhibitors

No antiviral activity against SARS-CoV-2 was demonstrated for the HIV protease inhibitors darunavir [39], amprenavir, saquinavir, neither indinavir on Vero E6 and Vero E6/TMPRSS2 [38,81], although indinavir has been predicting to bind with the SARS-CoV-2 PLpro [72,85]. Clinical efficacy of a 5-day treatment by the combination Darunavir/cobicistat was assessed and did not show any benefit compared to the standard of care [86]. Adverse events are controversial according to the studies, one of them showing that it was associated with negative outcomes in HIV-negative patients with severe COVID-19 pneumonia [86,87].

#### Other coronaviral protease inhibitors

Several protease inhibitors, some of them that had been co-crystallized with SARS-CoV-2 Mpro, harbor anti-SARS-CoV-2 activity. Among them, α-ketoamide inhibitors [88] exhibit an anti-SARS-CoV-2 activity at a MOI = 0.005 on Huh7 cells with a EC_50_ varying from 0.0004 μM and 0.02 μM with very high SI, while the antiviral activity was moderate on Vero cells with EC_50_ ranging from 5 to 13 μM [88].

#### Other antivirals

##### Daclatasvir

Daclatasvir is an inhibitor of the NS5a protein of HCV used in combination with other direct acting antiviral agents in HCV-induced hepatitis. The EC_50_ of daclatasvir against SARS-CoV-2, tested on three different cell lines (Huh7, Vero E6 and Calu3), with an end point assessment at 24 or 48 h by infectious virus evaluation was low, varying from 0.6 to 1.1 μM at an MOI of 0.1 or 0.01 [69].

##### Umifenovir

Umifenovir (Arbidol^®^) is an anti-influenza molecule used in China and Russia that may act as an entry inhibitor of SARS-CoV-2, by blocking trimerization of the spike protein and host cell adhesion [89]. Its antiviral activity has been demonstrated on Vero E6 cells with an EC_50_ comprised between 4.1 μM and 11 μM for a MOI of 0.05 and 0.002, respectively [22,90]. In a retrospective study carried out in a non-intensive care unit in China, it was not associated with improved outcomes [91,92].

### Antiparasitic agents

#### Antimalarial drugs

##### Quinolines

Quinolines, including chloroquine, amodiaquine, quinine, mefloquine, lumefantrine, piperaquine, pyronaridine and tafenoquine, are the most recommended drugs in the treatment of malaria. Chloroquine and hydroxychloroquine are also indicated in the treatment of some autoimmune diseases.

The median effective concentration (EC_50_) of chloroquine on SARS-CoV-1 on Vero E6 cells [93–96], ranged from 4.1 to 8.8 μM. The selectivity index, i.e., the ratio between the 50% cytotoxicity concentration (CC_50_) and EC_50_, was assessed in two assays and provided medium values of >11 and >31 [94,96]. The EC_50_ of chloroquine on MERS-CoV infected Vero E6 cells was 16 μM and 6.3 μM at MOI 0.01 and 0.1, respectively [93,97].

SARS-CoV-2 was also susceptible to chloroquine and hydroxychloroquine. *In vitro* effects of chloroquine on infected Vero E6 cells showed discrepant EC_50_ ranging from 0.1 to >50 μM with a MOI of 0.01 [80,98]. Wang *et al.* found an EC_50_ of 1.1 μM and a SI >88.5 at a MOI of 0.05 [21]. Gendrot *et al.* estimated the EC_50_ at 2.1 μM and a SI >47 at a MOI of 0.25 [99]. Liu *et al.* demonstrated that EC_50_ increased when the MOI increased [100]. These concentrations under 10 μM are higher than standard therapeutic plasmatic and lungs concentrations. For example, the plasmatic concentration observed during chloroquine prophylaxis given at 100 mg/day, ranges from 0.01 to 0.4 mg/l, i.e., 0.03 to 1.25 μM [101]. Moreover, the excellent tissue distribution of chloroquine leads to 200–700-fold blood concentration in the lung (lung concentration can reach up to 280 mg/kg) [102]. The EC_50_ of hydroxychloroquine assessed on SARS-CoV-2 infected Vero E6 cells ranged from 1.5 μM to 17.3 μM [22,98] and from 0.7 to 6.3 μM on Vero cells [103]. These concentrations were consistent with concentrations observed in human plasma and lungs. An oral uptake of 400 mg of hydroxychloroquine led to a maximum blood concentration (C_max_) of 1.22 μM [104]. Moreover, hydroxychloroquine accumulated 30× more in the lungs than in the blood (around 0.3 μM vs 7.8 μM at 6 h) [105]. However, one study using as cell support TMPRSS2 (transmembrane serine protease 2) expressing human lung cell line (Calu-3) and TMPRSS2-Vero resulted in the absence of antiviral efficacy of chloroquine against SARS-CoV-2 [83]. Another work also described the absence of an antiviral effect of hydroxychloroquine on SARS-CoV-2 at a MOI = 0.1 on infected human airway epithelia reconstituted from human primary cells obtained from nasal or bronchial biopsies [106].

Chloroquine and hydroxychloroquine are understood to act on the viral post-entry step. Viral entry occurs after the interaction between the S1 subunit with the ACE2 cell surface receptor and a cleavage at the S1/S2 junction by TMPRSS2. This leads to an interaction with cell surface phospholipid bilayers. The nucleocapsid of the virus then gets into endosomal vesicle. After acidification of the late endosome, the cathepsin enables the release of viral genomic RNA. Cathepsin B and cathepsin L are endosomal cysteine proteases, whose activation requires a low pH [107], prevented in this case by chloroquine or hydrochloroquine. It has been demonstrated that both molecules could alter the function of lysosomes, by increasing their pH, while late endosome cathepsins are active at low pH only. This mechanism has been well demonstrated in the SARS-CoV-2 infection for hydroxychloroquine. The two molecules may also inhibit the terminal glycosylation of ACE2, and thus viral entry in cells [21,95,100]. The spike protein of SARS-CoV-2 used the ACE-2 receptor for entry, but also sialic acids and gangliosides. *In silico* analyses showed that the viral spike protein was not able to bind gangliosides in the presence of chloroquine or hydroxychloroquine [108]. Moreover, chloroquine could bind to the SARS-CoV-2 PLPro [109]. The use of chloroquine has been proposed as a treatment for COVID-19 early in the pandemics, especially in a Chinese study that concluded to benefits in clinical outcome and viral clearance [110]. Hydroxychloroquine, that harbors a better safety profile than chloroquine, has also been used both for treatment and as post-exposition prophylaxis, with a lot of discrepant results, and controversies. Gautret *et al.* showed a faster viral clearance in treated patients comparatively with the control groups [111]. A systematic review and meta-analysis of five randomized clinical trials including 5577 patients treated with hydroxychloroquine or placebo/standard-of-care for pre-exposure prophylaxis, post-exposure prophylaxis, or outpatient therapy for COVID-19, found that the hydroxychloroquine was associated with a 24% reduction in COVID-19 infection, hospitalization or death, with no serious adverse events reported [112]. Inversely, others have shown that hydroxychloroquine was not associated with any benefits in the COVID-19 treatment, neither as a post-exposure prophylaxis [113–115]. In addition, Yang *et al.* reanalyzed data from two previous randomized controlled trials assessing the efficacy of hydroxychloroquine as post-exposure prophylaxis, one showing the absence of any efficacy [116], the other one suggesting that a very early use was associated with an increased protection against the infection [117]. The authors conclude, after reanalysis, that hydroxychloroquine is beneficial in postexposure prophylaxis. Another study showed that hydroxychloroquine may reduce COVID-19 by as much as 65% when given within 3 days of exposure [118]. With a lot of discrepant results on clinical studies probably due to the absence of standardization in the protocol of use, the heterogeneity in patients included, these results remain very conflicting [119]. However, global safety profile is acceptable with gastro-intestinal troubles reported as the most frequent adverse events, and without any serious cardiac adverse event when monitoring is carried out [113].

It is notable that methylene blue also exhibited an *in vitro* anti-SARS-CoV-2 activity with a mean EC_50_ of 0.30 μM at a MOI of 0.25 [99].

Amodiaquine revealed an anti-SARS-CoV-1 activity on Vero E6 and Vero 76 and an anti-SARS-CoV-2 activity on Vero, Vero E6 [20,93,98,120]. It is notable that in one study, the EC_50_ assessed on Calu-3 cells was found to be >50 μM i.e., ten-times higher than in Vero cells [36]. Desethylamodiaquine, the metabolite of amodiaquine, showed high *in vitro* efficacy with an EC_50_ of 0.52 μM and a SI of 166 [99]. A fixed dose of artesunate-amodiaquine (200 mg/540 mg), the dose recommended in malaria treatment, led to a plasma C_max_ of desethylamodiaquine around 879 ng/ml (around 4 μM) [121]. About 0.07% of the administered oral dose (8.6 mg/kg) of amodiaquine was found in rat lungs [122].

Mefloquine is a blood schizonticide from the class of methanolquinolines effective against *Plasmodium falciparum* and *Plasmodium vivax*. The EC_50_ of mefloquine against SARS-CoV-2 varied between 1.8 and 8.1 μM on Vero E6 cells (MOI 0.002 to 0.25) with low selectivity indexes between 2.3 and 8 [98,123], while on Calu-3 cells no antiviral activity was observed [36]. At 10 μM, mefloquine completely inhibited the cytopathic effect on Vero E6 cells infected by SARS-CoV-2 [124]. Mefloquine administered at the malaria therapeutic dose (1250 mg) led to a blood concentration of 1648 ng/ml (around 4 μM) in healthy males [125]. A study on post-mortem cases showed that mefloquine levels are ten-times higher in the lung than in the blood (a concentration which can go up to 180 mg/kg in the lung) [126].

The antiviral activity of pyronaridine was previously demonstrated against the Ebola virus and more recently, against the SARS-CoV-2 [29,127]. Bae *et al.* showed that pyronaridine at a MOI = 0.01 could inhibit SARS-CoV-2 replication in Vero cells with an EC_50_ of 1.1 and 2.2 μM, after 24 and 48 h of culture, respectively. Gendrot *et al.* showed that pyronaridine exerted effective anti-SARS-CoV-2 activity in Vero E6 cells at a MOI of 0.25 after 48 h of contact with an EC_50_ of 0.72 μM and a SI of 22 [99]. Pyronaridine tetraphosphate given at 720 mg day led to a plasma concentration of 271 ng/ml (around 0.3 μM) in humans and a t_1/2_ of 33.5 days [128]. A single oral dose of 2 mg (10 mg/kg) in rats led to a blood C_max_ of 223 ng/ml and a lung C_max_ of 36.4 μg/g (165 more concentrated) [129]. The anti-SARS-CoV-2 activity of pyronaridine is compatible with malaria oral therapeutic doses.

Quinine, the second-line treatment for severe malaria after artesunate IV, showed medium anti-SARS-CoV-2 *in vitro* activity with an EC_50_ of 10.7 μM and an EC_90_ of 38.8 μM [130]. A 600 mg single oral dose of quinine sulphate led to blood C_max_ around 3.5 mg/l (around 8.5 μM) [131]. However, after intravenous doses of 10 mg/kg of quinine in rats, the observed concentration of the lung/blood ratio was 246 [132]. The *in vitro* effectiveness of the concentration in the lungs to cure SARS-CoV-2 is achievable in humans.

Halofantrine, harbors an anti-SARS-CoV-2 activity, evaluated by viral expression (GFP) with a MOI = 2.2 with an EC_50_ = 0.3 μM on HeLa ACE2 [26].

The maximal plasmatic concentration of tafenoquine, used for *Plasmodium vivax* infections, exhibited interindividual variability, its bioavailability being greatly influenced by high-fat meals. Preliminary studies demonstrated that tafenoquine had an anti-SARS-CoV-2 activity in Vero E6 cells in two studies that found an EC_50_ from 2.5 to 16 μM [133,134].

##### Artemisinin & derivatives

Artemisinin extracted from the wormwood *Artemisia annua*, harbors a potent activity against *Plasmodium*, the anti-SARS-CoV-2 activity of *Artemisia annua* derivatives has been explored in cell cultures [127,135,136]. Gilmore *et al.* showed that artesunate was more potent than the *Artemisia annua* plant extracts, artemisinin and artemether (which was found not to be effective against the virus) with an EC_50_ of 7 μg/ml (3.4 μM), 128–260 μg/ml (7.3 μM), 151 μg/ml (535 μM), and >179 μg/ml (>600 μM) respectively on Vero E6 cells, and similar results on human hepatoma Huh7.5 cells. It is notable that close to complete inhibition of the viral replication was obtained for 15 μg/ml and 22 μg/ml on Vero E6 and Huh 7.5 cells, respectively. However, dihydroartemisinin is the active metabolite of all artemisinin derivatives (artemisinin, artemether, artesunate). Dihydroartemisinin showed low anti-SARS-CoV-2 activity with an EC_50_ of 20.1 μM at an MOI of 0.25 [130]. Artesunate, which displays a better oral bioavailability compared to artemether [137], with a maximal plasmatic peak concentration of 29.5 μM after an IV bolus of 120 mg and 2.6 μM after an oral dose of 100 mg, could be an effective antiviral *in vivo* [138].

For the treatment of uncomplicated malaria due to *P. Falciparum*, since 2002, international guidelines from the World Health Organization recommend using artemisinin-based combination therapy. A study showed that concentrations of fixed-doses of artemisinin-based combination therapy equivalent to C_max_ of the two partners at commonly recommended doses for uncomplicated malaria, were able to inhibit 27.1 to 72.1% of the Vero E Cells infected with SARS-CoV-2 [139]. Treatment with artesunate-mefloquine (expected blood C_max_ at 8.3 and 1 μM) leads to replication inhibition of 72.1%.

#### Other antiparasitic agents

##### Nitazoxanide

Nitazoxanide is a broad spectrum anti-infective drug that belongs to the class of thiazolides. It was approved in 2002 for the treatment of *Cryptosporidium* and *Giardia lamblia* infections. Its antiviral activity was previously shown *in vitro* against a wide range of RNA and DNA viruses including Influenza virus, Respiratory Syncytial Virus, rotavirus, Hepatitis B virus, Hepatitis C virus, the dengue, the Human Immunodeficiency Virus, and more recently against SARS-CoV-2 [140]. Indeed, in one study, its EC_50_ was 2.1 μM on Vero E6 cells with a high selectivity index (>17) [21]. One pharmacokinetic analysis suggested that nitazoxanide plasma levels could reach concentrations above the reported EC_50_ level [43]. As of July 2021, it is being studied in 30 ongoing or planned clinical trials, both as a treatment and as a prophylaxis. A single 500 mg dose treatment of nitazoxanide reaches within 1–4 h to tizoxanide (active metabolite of nitazoxanide) plasma concentrations greater than 10 μM, with a half-life of 1.3–1.8 h and good tolerance [141]. Nitazoxanide 600 mg BID for 7 days has been evaluated in a randomized, double-blind pilot clinical trial versus Placebo among 50 hospitalized patients (25 in each arm) with mild respiratory insufficiency due to SARS-COV-2 infection (ClinicalTrials.gov NCT04348409) [142]. Nitazoxanide showed superiority for the mean time of hospital discharge arm (6.6 vs 14 days, p = 0.021) and of negativation of the RT-PCR. In addition, inflammatory markers were significantly lower in the nitazoxanide arm. Moreover, even if the difference was not significant, it should be noted that six patients died in the placebo arm compared to only two patients in the treatment arm.

##### Niclosamide

Niclosamide is an anthelmintic developed in 1953 by Bayer laboratories, approved by the FDA in 1982 for human use, and currently included in the World Health Organization's list of essential medicines. It acts as an anticestodal by uncoupling the oxidative phosphorylation, thus inhibiting the production of ATP, which is essential for the energetic metabolism of the parasite [143]. It is effective against *Taenia saginata* (beef tapeworm), *Taenia solium* (pork tapeworm), *Diphyllobothrium latum* (fish tapeworm) and *Hymenolepis nana*. Drug repurposing screening studies identified niclosamide as a multifunctional drug that displays a large range of clinical applications such as bacterial and viral infections, metabolic diseases, neuropathic pain, rheumatoid arthritis and even cancer. Among the proposed mechanisms of action, it may regulate several signalling pathways and biological processes including notably mTOR (mammalian target of rapamycin), STAT3 (signal transducer and activator of transcription 3), and NF-κB (nuclear factor κ-light-chain-enhancer of activated B cells) signalling pathways, and may also reduce endosomal acidification and viral dsRNA replication [144,145]. Its antiviral activity against SARS-CoV-1 was demonstrated on Vero E6 cells [146,147] and against SARS-CoV-2 on the same cell lines and also Huh7 and Vero [6,20,25,148].

##### Ivermectin

Ivermectin is a semisynthetic broad spectrum anthelmintic agent, that is orally administered in the treatment of intestinal strongyloidiasis due to *Strongyloides stercoralis*, onchocerciasis due to *Onchocerca volvulus* and scabies due to *Sarcoptes scabiei*. It showed an antiviral activity against SARS-CoV-2 at a MOI of 0.01, on Vero E6 cells with an EC_50_ of 1.7 μM evaluated by viral expression (N protein) [149]. Congruently, computational studies predicted that ivermectin could dock in two specific regions of the SARS-CoV-2 spike and of the ACE2 receptor, with a binding energy with the complex spike-ACE2 of -18 kcal/mol [150]. A systematic review and meta-analysis of 15 trials showed that ivermectin reduced risk of death and that ivermectin prophylaxis could reduce SARS-CoV-2 infection by 86% with low-certainty evidence. While ivermectin did not show any benefit in reducing ‘need for mechanical ventilation’, ivermectin use was more often associated with ‘improvement’ than ‘deterioration’ with no difference in occurrence of severe adverse events [151].

##### Emetine

Emetine is a toxic alkaloid of ipecac, extracted from the root of the plant *Psychotria Ipecacuanha* (Rubiaceae) used in phytomedicine for centuries and known to be the main component of ipecac syrup used as an emetic. In eukaryotic cells, emetine irreversibly blocks ribosome movement along the mRNA thus preventing protein synthesis strands and inhibiting DNA replication. It has also been shown that it may up- and down-regulate several genes [152]. It also exhibits many biological properties including antimalarial, antineoplastic, antiamoebic, contraceptive and antiviral activities against vaccinia, dengue, Zika, Ebola and SARS-CoV-2 viruses [19,152–156]. It was reported that emetine could inhibit dengue virus at an early stage of the replication cycle, possibly by blocking the translation of the polyprotein precursor, a key step for the formation of viral proteins and further RNA replication [153]. It also inhibits Zika and Ebola virus infections by inhibiting viral replication and decreasing viral entry [155]. It is no longer currently marketed in medical specialties, but has long been used in the past for the treatment of intestinal amebiasis and for emptying the stomach in the cases of acute intoxication.

Adverse events after its ingestion include cardiac and hepatic events, renal toxicity, diarrhoea and vomiting. Emetine has been shown to inhibit SARS-CoV-1 and SARS-CoV-2 *in vitro*, with EC_50_s <1.0 μM in Vero cells and in Caco2 cell lines (EC_50_ = 0.05 μM for SARS-CoV-1 and 0.5 μM for SARS-CoV-2). Interestingly, 8 and 24 h after oral administration, the concentration of emetine in the lung is ∼173 and ∼294× higher than in plasma [154].

### Antibiotics

#### Clofazimine

Clofazimine is a highly lipophilic antimicrobial agent that acts on the respiratory chain of bacteria and on ion transporters. By oxidizing the reduced form of clofazimine, the intracellular cycle of redox reactions provides ROS with an antimicrobial activity. Moreover, clofazimine interacts with the phospholipid bilayer of the membrane, generating antimicrobial lysophospholipids that favor membrane dysfunction, which raises anomalies in potassium (K^+^) recapture. Clofazimine is used in the treatment of leprosy. It also displays an anti-inflammatory activity due to the suppression of T-lymphocyte activity. The main adverse event is the orange-pink to brownish-black discoloration of the skin, conjunctivae, and body fluids. Antiviral activity has been observed on SARS-CoV-2 on Huh7, Vero E6 with a low EC_50_ ranging from 0.08 to 0.5 μM [25,157].

Yuan *et al.* also demonstrated this antiviral effect on primary cells. First, on human embryonic stem cell-derived cardiomyocytes, clofazimine at 10 μM could reduce viral titers in the cell lysate by >3-log10 compared with the DMSO control. Second, an *ex vivo* lung culture system infected with SARS-CoV-2 for 24 h and clofazimine treatment starting 2 h post-inoculation revealed a potent inhibition of viral replication [5]. In this study, the authors conclude that the effective antiviral concentration of clofazimine of 310 nM may be achievable in patients, the peak serum concentration being 861 nM.

A docking study using a virtual screening procedure of the 1615 FDA-approved drugs selected clofazimine as one of the top 25 compounds with lowest docking score with SARS-CoV-2 main protease [158].

#### Azithromycin

Azithromycin is a broad-spectrum macrolide antibiotic, approved by the FDA in 1991. As is the case for other macrolides, it inhibits bacterial protein synthesis and translation, in addition to an additional immunomodulatory effect [159]. It has an extensive uptake in tissue, particularly in the lung, tonsils and prostate [159]. It exhibits an antiviral activity against SARS-CoV-2 with an EC_50_ of 2.1 μM on Vero E6 cells at a MOI of 0.002 [22]. It is notable that the combination of hydroxychloroquine at 5 μM and azithromycin at 5 μM tested on Vero E6 cells resulted in a relative inhibition of SARS-CoV-2 of 97.5% [160]. It has been predicted that azithromycin could interact with a conserved amino acid triad Q-134/F-135/N-137, located at the tip of the SARS-CoV-2 spike protein, but also displays strong interactions with the viral main protease, and two host proteins involved in the replication cycle of the SARS-CoV-2: the receptor ACE2 and the host cathepsin L [161,162].

#### Doxycycline

Doxycycline, a second-generation tetracycline with broad-spectrum antimicrobial, antimalarial and anti-inflammatory activities, showed an EC_50_ of 4.5 μM against SARS-CoV-2-infected Vero E6 cells at a MOI of 0.25 [163]. Doxycycline may inhibit SARS-CoV-2 entry and post-entry steps in Vero E6 cells. C_max_ value in healthy volunteers of doxycycline reaches 1.7 and 5 μg/ml (around 3.4 and 10 μM) after daily per os doses of 100 mg or 200 mg [164,165]. The C_max_/EC_50_ and C_max_/EC_90_ ratios for doxycycline in plasma ranged from 0.75 to 2.21. The C_max_/EC_50_ ratios in plasma would appear low to reach effective concentrations to inhibit SARS-CoV-2 in humans. However, in the lung, doxycycline was two to four-times higher than in plasma [166] as shown by the C_max_ value from 3.4 to 20 μg/g observed in the lung after the uptake of the same dosing of 100 or 200 mg.

Computational approaches showed congruently that doxycycline could inhibit SARS-CoV-2 entry and viral replication stages, by binding to the spike protein [167]. In addition, the SARS-CoV-2 main protease (M^pro^ or 3C-like protease) has also been predicted as a possible target for doxycycline and, broadly, tetracyclines [168,169].

#### Teicoplanin

Teicoplanin, a glycopeptide antibiotic used to treat Gram-positive bacterial infections prevents the early step of the viral life cycle by inhibiting cathepsin L in the late endosome/lysosome and blocking the entry of pseudo-typed viruses for Ebola, MERS-CoV and SARS-CoV-1 [170]. For SARS-CoV-2, docking studies also showed that this molecule harbored a relatively high affinity with the 3CL^Pro^ protease, with ten to twenty-times greater potency in inhibiting protease activity than other drugs such as atazanavir, chloroquine, hydroxychloroquine, azithromycin, or lopinavir [171]. To date, the antiviral activity on SARS-CoV-2 has only been assessed with spike-pseudoviruses, and shows an IC_50_ of only 1.66 μM, which is lower than the routine serum drug concentration (∼7–8 μM) [172]. Further *in vitro* studies are needed to assess the antiviral activity of this molecule on SARS-CoV-2.

#### Clofoctol

Clofoctol exhibits an important antiviral activity in Vero 81 cells against SARS-CoV-2, by blocking translation initiation of viral RNA at a post-entry step. Interestingly, the pulmonary peak concentration of clofoctol can reach more than 20× higher than the concentration required to inhibit by 95% the viral in human pulmonary cells. Moreover, it allowed a decrease in viral load, a reduction in inflammatory gene expression and an improvement in pulmonary pathology in mice [173].

### Antipsychotics

### Phenothiazine derivatives

Promazine is a dopaminergic antagonist, a H1 receptor antagonist, a muscarinic antagonist, and a serotonergic antagonist. It is a phenothiazine antipsychotic drug with antiemetic properties and acts as a prolyl oligopeptidase inhibitor. It does not exhibit any antiviral efficacy against SARS-CoV-1 on Vero 76 cells with EC_50_ ranging from 7.4 to 28 μM [63,174].

Chlorpromazine is an antipsychotic agent with anti-emetic activity. It antagonises dopamine receptors. It is active against SARS-CoV-1 on Vero E6 cells [93,94] and also recently showed an antiviral effect against SARS-CoV-2 [98]. A sensitivity assay against SARS-CoV-2 on Vero E6 found an EC_50_ between 3.1 μM with an evaluation by CPE and a MOI of 0.004 [98]. In addition, the EC_50_ on MERS-CoV-infected Huh7 cells and MDM cells were 4.9 and 14 μM with MOI of 0.005 and 0.1, respectively [94,175].

### Thioxanthene derivatives

Chlorprothixene is a dopamine receptor antagonist (D1, D2, D3) used as an antipsychotic drug. Chlorprothixene also strongly blocks the 5-HT2, histamine H1, muscarinic and α1 adrenergic receptors. It has shown antiviral activity against SARS-CoV-2 with an EC_50_ of 8.9 μM on Vero E6 at a MOI of 0.002 [176].

#### Antihistaminics

### Desloratadine

Desloratadine is synthetic piperidinyl-benzimidazole derivative, and a reversible competitive inhibitor of histamine H1 receptors, with antiallergic properties. It inhibits SARS-CoV-2 replication at a MOI of 0.5 on A549/ACE2 cells with an EC_50_ of 0.9 μM, evaluated by viral expression of the S protein [30].

#### Ebastine

Ebastine is a second-generation piperidine H1 antihistamine which potently antagonizes H1 histamine receptors. It demonstrates an antiviral activity against SARS-CoV-2 with an EC_50_ ranging from 1.2 to 6.9 μM on four different cell lines: Calu3, Huh7.5, Vero and Vero CCL81 [20,29].

#### Kinase inhibitors

Tyrosine kinase inhibitors are orally-administered targeted treatment of malignancies. They are competitive inhibitors of ATP at the catalytic binding site of tyrosine kinase. They target different kinases, and cause skin toxicity, mainly folliculitis, in addition to myelosuppression (anaemia, thrombopenia, neutropenia) and compound specific adverse events.

Imatinib was the first labelled tyrosine kinase inhibitor, indicated for chronic myeloid leukemia with the Philadelphia chromosome [93,177]. On SARS-CoV-1 and MERS-CoV-infected Vero E6 cells, the EC_50_ ranged from 5 to >20 μM. A lower EC_50_ (3.2 μM) was observed for SARS-CoV-2 on the same cells in two different assays with a MOI of 0.004 and 0.01 [98].

Lapatinib is used to treat breast cancer with an overexpression of ErbB2 receptors. It showed an antiviral effect on SARS-CoV-2 at a MOI = 0.5 on A549/ACE2 cells with an EC_50_ of 1.6 μM [30].

Dacomitinib is indicated for the treatment of metastatic non-small cell lung cancer. On Huh7.5 cells, with an MOI of 1, an EC_50_ against SARS-CoV-2 was 0.8 μM when the effect was evaluated by visual inspection of cytopathic effects, with a selectivity index of 15 [29]. On Calu-3 and with a MOI of 0.5, the EC_50_ was 0.04 μM, with a high selectivity index of 226 and an evaluation by visual inspection of cytopathic effects [29].

Bosutinib is indicated for Philadelphia + chronic myeloid leukemia. On Huh7 cells, SARS-CoV-2 at a MOI of 0.2 was inhibited by Bosutinib with an EC_50_ of 0.02 μM with a high selectivity index >100 [25].

Fedratinib, used for treatment of myelofibrosis, exhibits an antiviral activity against SARS-CoV-2 at a MOI of 0.2 on Huh7 cells with an EC_50_ of 0.02 μM and a selectivity index of 83 [25].

Gilteritinib is indicated for the treatment of acute myeloid leukemia with FLT3 receptor mutation. FDA approved in 2018 [20,25,36], it shows a heterogenous profile of activity against SARS-CoV-2 depending on the cells used in the different sensitivity assays. The most potent activity was observed on Huh7 cells, at an MOI = 0.2 with a low EC_50_ of 0.2 μM and a selectivity index of 8.9. Intermediately, on Vero cells with a MOI = 0.01, the EC_50_ was 6.8 μM with a selectivity index of 5.5. Finally, on Calu-3 cells, at a MOI of 0.1, no antiviral activity could be observed with an EC_50_ >50 μM.

Nilotinib is a Bcr-abl kinase inhibitor that was approved by the FDA in 2007 for the treatment of Ph+ chronic myeloid leukemia. Adverse events include myelosuppression and prolongation of the QT interval. Interstitial pneumonia has also been reported. It seems to have a potent antiviral activity against SARS-CoV-2 at a MOI = 0.1 with an EC_50_ of 0.08 μM on Vero E6 cells [178] and <0.01 μM on Vero cells with a selectivity index ≈3000 [179]. This antiviral activity operates by an unknown mechanism. A virtual screening of the ZINC database showed that nilotinib could interact with the NSP12-NSP7-NSP8 interface of SARS-CoV-2, which is the essential component of the replication complex of SARS-CoV-2 with NSP12 consisting in the catalytic subunit with RNA-dependent RNA polymerase activity, and NSP7 and NSP8 being cofactors that stimulate this polymerase activity [180]. Another molecular docking study that screened 15,000 molecular candidates from DrugBank and natural compounds from the Traditional Chinese Medicine Systems Pharmacology Database showed that nilotinib was among the top ten compounds binding to the RBDs of the viral spike with a free energy of -7.9 kcal/mol [181].

### Immunosuppressive agents

Mycophenolate is an immunosuppressant and antiproliferative drug used to treat prophylaxis or organ rejection in renal transplant recipients in combination with cyclosporin and corticosteroids. It acts as a selective and competitive inhibitor of inosine monophosphate dehydrogenase and thus inhibits *de novo* guanosine nucleotide synthesis. It also displays a potent inhibitory effect on proliferative T and B lymphocytes responses. It has also been shown that it could inhibit TMPRSS2, which is involved in SARS-CoV-2 entry. Molecular docking showed that it could bind to the active site of SARS-CoV-2 PLPro, and thus inhibit the viral replication [85]. On SARS-CoV-2-infected Vero E6/TMPRSS2, an EC_50_ of 0.9 μM was found at a MOI of 0.01 [182]. The EC_50_ of mycophenolate activity assessed on MERS-CoV-infected Vero E6 and Vero cells was 1.5 μM with a MOI 0.01 and 0.5 μM at MOI 0.001 [97,183].

Cyclosporin is a calcineurin inhibitor which was FDA-approved in 1983, with potent immunosuppressive properties on T cells for preventing organ rejection and preventing and treating graft versus host disease in bone marrow transplantation. Its immunomodulatory properties are also used for various autoimmune conditions such as rheumatoid arthritis, and inflammatory diseases such as severe psoriasis. Its antiviral effect explored on SARS-CoV-2-infected Calu-3 cells showed an EC_50_ of 0.2 μM with a MOI of 0.5 [29] and an EC_50_ of 4.7 μM with a MOI of 0.1 [36]. The highest EC_50_ was observed on Vero cells and was 5.8 μM at a MOI of 0.01 with an evaluation by viral expression of N protein [20].

Although immunosuppressant drugs are not an option for therapeutic use in COVID-19, it is worthwhile exploiting the antiviral profile that has been shown *in vitro*, in organ transplant recipients receiving long term treatment with these molecules.

### Cardiac glycosides

Cardiac glycosides are organic compounds that potently inhibit the Na+/K+ exchanging ATPase, leading to the increase of Na+ intracellular concentration, and to an increase in intracellular Ca2+ via the Na+/Ca2+ pump. This increased intracellular calcium concentration is the basis of the inotropic property of these drugs. Although they cannot reasonably be an option in the treatment of COVID-19, some sensitivity assays have been performed to assess their potential antiviral activity.

Digoxin originated from *Digitalis purpurea* is indicated for atrial fibrillation and heart failure. It has been shown to have an antiviral activity against SARS-CoV-2 with an EC_50_ ranging from 0.04 (at a MOI of 0.1) to 0.2 μM (at a MOI of 0.01) on Vero cells [20,179].

Digitoxin has a longer half-life than digoxin and is used for congestive cardiac insufficiency, arrythmias and heart failure. EC_50_ for SARS-CoV-2 ranges from 0.1 (at a MOI of 0.1) to 0.2 μM (at a MOI of 0.01) on Vero and Calu-3 cells [20,179,184].

Ouabain is a glycoside obtained from the seeds of *Strophanthus gratus* and is indicated for atrial fibrillation flutter and heart failure, with a potent antiviral activity against SARS-CoV-2 at a MOI of 0.01 with an EC_50_ <0.1 μM on Vero and Calu-3 cells with high selectivity indices [20,185].

### Antineoplastics

Antineoplastic agents do obviously not represent a clinical option for SARS-CoV-2 infection, however their potent antiviral activity shown in a few *in vitro* studies could be interesting to explore in patients currently treated by these molecules.

Gemcitabine is a cytidine analog that blocks the enzyme that converts cytosine into deoxycytosine. It also blocks thymidylate synthetase, resulting in blocking DNA replication and in premature apoptosis and arrested tumor growth. It is labelled as a chemotherapeutic agent in various carcinomas. Its antiviral activity against SARS-CoV-2 was assessed at a MOI of 0.005 on Vero E6 cells at an EC_50_ of 1.2 μM with a selectivity index >32 [186].

Thioguanine is a 6-thiopurine analog, competing with hypoxanthine and guanine, and also belongs to the family of antimetabolite agents. It is used in acute non-lymphocytic leukemias. It displays an additional cytotoxic action due to its incorporation into RNA. On SARS-CoV-2 infected Huh7 cells, at a MOI of 0.2, the EC_50_ of thioguanine was 0.2 μM with a selectivity index >9.3 [25]. It inhibits the viral PLPro and this activity has been previously demonstrated too on SARS-CoV-1, MERS-CoV by biochemical assays but not in culture cell assays [187].

### Anti-estrogens

Tamoxifen is a non-steroidal anti-estrogen compound that competitively inhibits estrogen binding to its receptor and is used for the treatment of estrogen receptor-positive breast cancer. It was FDA-approved in 1977.

Toremifene is a non-steroidal selective estrogen receptor modulator with a structure that is closely related to tamoxifen and which is also indicated in the treatment of breast cancers. Tamoxifen citrate and toremifene were respectively tested in three and four sensitivity assays against SARS-CoV-2 with EC_50_ ranging from 1.8 to 34 μM and 4.8 to 12 μM on Vero E6, respectively [20,93,98,176]. It should be noted that tamoxifen citrate does not exhibit any antiviral effect against SARS-CoV-1 with an EC_50_ of 93 μM on Vero E6 cells [93]. However, the EC_50_ of raloxifene against SARS-CoV-2 was 3.8 μM on A549/ACE2 at a MOI of 0.5 [30].

### Calcium channel blocker with an action on the cardiovascular system

Amlodipine is an antihypertensive drug belonging to the family of dihydropyridine calcium channel blockers, used for the treatment of hypertension, coronary artery disease and chronic stable angina. It does not exhibit any antiviral activity as assessed in six cell culture assays with an imprecisely defined EC_50_, but <50 μM [188] on Vero E6 and Calu-3 cells, and <10 μM on HPSC-derived organoids and A549/ACE2 [189], moreover with low selectivity indexes.

Verapamil is an old drug belonging to the family of non-dihydropyridine calcium channel blockers such as diltiazem, for which an antiviral activity was demonstrated in combination with remdesivir in one study [48]. It is used for hypertension, arrythmias and angina. On SARS-CoV-2-infected Huh7 cells (MOI 0.2), the EC_50_ of verapamil was low, at 0.5 μM with a selectivity index of >3.8 μM [25].

### Drugs acting on the alimentary tract & metabolism

Loperamide is a long-acting synthetic antidiarrheal by inhibiting peristaltic activity. It is an opioid μ receptor agonist and a non-selective calcium channel blocker. Although it is not absorbed from the gut, its antiviral affect was assessed against SARS-CoV-2 and revealed EC_50_ varying from 9.3 μM at a MOI of 0.01 [20] on Vero cells to 13 μM at a MOI of 0.1 on Calu-3 cells [36]. Molecular docking has also predicted that it could bind to the SARS-CoV-2 PLPro with a docking-free energy of -7.1 kcal.mol^-1^ [72,85].

Metoclopramide is a dopamine D2 antagonist with prokinetic and antiemetic effects that treats nausea, vomiting and gastro-esophageal reflux disease. It was FDA approved in 1980. On Huh7 cells, it shows anti-SARS-CoV-2 activity at a MOI of 0.2 with an EC_50_ of 0.5 μM [25].

### Glycosaminoglycan attachment inhibitors

Anticoagulants such as the defence iron-binding protein, lactoferrin naturally present in exocrine secretion or heparin used to impede the COVID-19-induced hypercoagulability have been assessed for their anti-SARS-CoV-2 activity. They inhibit the attachment of viruses to glycosaminoglycan of the surface cells, reaching to an inhibition of the viral entry. Heparin harbored an *in vitro* anti-SARS-CoV-2 activity with an EC_50_ of 2 μM on Vero E6 cells, that is compatible with standard therapeutic concentrations [190]. A study carried out in 17 hospitals in Spain showed that heparin used in 1734 patients, was associated with lower mortality even when saturation of oxygen was <90%, and temperature >37°C [191]. Heparin also offers the advantage to have anti-inflammatory effects, and has been associated with an increased lymphocyte count and decreased IL-6 levels compared with control group [192].

### Others

Lomitapide is a microsomal triglyceride transfer protein inhibitor indicated for homozygous familial hypercholesterolemia, largely used to decrease LDL-cholesterol and total cholesterol levels. Anti-SARS-CoV-2 activity at a MOI of 0.2 was shown on Huh7 cells, with an EC_50_ of 0.5 μM, efficacy being evaluated by the viral expression of N protein [25].

Aprotinin, a single-chain polypeptide isolated from bovine lung with antifibrinolytic and anti-inflammatory activities, camostat and nafamostat, two serine protease inhibitors, are three antifibrinolytic drugs tested in numerous sensitivity assays for SARS-CoV-2. Camostat and nafamostat may act by inhibiting the host TMPRSS2. Given the thrombotic risk in SARS-CoV-2 infection, these drugs, although they showed a potential antiviral activity especially for camostat and nafamostat with a respective EC_50_ ranging from 0.3 μM (on Calu-3) and >50 μM (on Vero) [20,29] and from 0.002 (on Calu-3) and >100 μM (on Vero E6/TMPRSS2) [193,194], cannot be considered as therapeutic options. Efficacy of camostat has anyway been assessed in early stages of infection (<5 days after symptoms onset) in adolescents and adults >= 18 year-old infected by SARS-CoV-2. Camostat treatment did not show any increase adverse events during hospitalization, but neither show a decrease in the mean time to clinical improvement, in the progression to intensive care unit, or mortality (ClinicalTrials.gov identifier: NCT04321096) [195].

Apilimod is an inhibitor of the production of IL-12 and IL-23, initially developed for the treatment of Crohn's disease and rheumatoid arthritis, but which was ultimately not effective in these indications. It also inhibits the lipid kinase enzyme PIKfyve. It was subsequently repurposed for Ebola virus disease and Lassa fever. It is not currently FDA-approved. The EC_50_ obtained on SARS-CoV-2-infected Vero E6 cells was 0.02 μM at a MOI of 0.002 with an evaluation of cytopathic effects and viral expression. Other sensitivity assays carried out on HeLa-ACE2, A549/ACE2, 293T/ACE2 and Huh-7/ACE2 showed low EC_50_ below 0.9 μM [23,26–28].

Auranofin that is a metallothiol-based drug used in rheumatoid arthritis, induces a 95% reduction in viral RNA at 48 h, in Huh7 cells, with a low EC_50_ at 1.4 μM, by inhibiting the SARS-CoV-2 PLPro [196]. Auranofin has also the advantages to harbor anti-inflammatory and anti-ROS properties interesting to exploit in the COVID-19.

Table 2 summarizes the main studies of sensitivity assays i.e., where at least three tests were performed for each compound, regardless of the virus. The main results of molecular docking studies are summarized in Supplementary Table 1.

Finally, when comparing the results of molecular docking with those of the most potent compounds found in *in vitro* sensitivity assays based on an EC_50_ <3 μM, 18 compounds were found by both approaches. Fourteen of these are currently FDA approved. These include digitoxin, a cardiac glycoside, three antivirals (remdesivir, nelfinavir and lopinavir), three antimalarial drugs (amodiaquine, chloroquine and hydroxychloroquine), one immunosuppressant (Cyclosporin A), two antineoplastics (nilotinib and tretinoin), one anti-inflammatory (celecoxib), two antibiotics (azithromycin and clofazimine) and one anthelminthic (ivermectin). As possible therapeutic options in COVID-19, when considering preliminary available results from clinical studies, the possibility of oral administration and the adverse events inherent to their pharmacological properties, nelfinavir, favipiravir, azithromycin, clofazimine, clofoctol, ivermectin, nitazoxanide, amodiaquine, heparin, chloroquine and hydroxychloroquine represent possible drug candidates for COVID-19 treatment.

## Discussion

In this study, we attempted to review as exhaustively as possible the different molecules that have been tested *in vitro* on SARS-CoV-2. In recent months, we have seen a competition between drugs recently produced by the pharmaceutical industry (lopinavir, remdesivir, ritonavir) and older molecules. It is clear that the significant paradigm that is emerging is that older molecules, known to be active in a certain area, are likely to be active in other functions. Thus, among the usable molecules that are effective *in vitro* on SARS-CoV-2, there are molecules belonging to therapeutic classes as different as anti-malarial drugs, other anti-parasitic agents, antibiotics, anti-psychotics, immunosuppressive agents, cardiotonic glycosides and many other families of molecules. Some of these molecules have been known for a long time, all of them are available and are extremely cheap, which poses the problem of managing therapeutic trials using these molecules in pathologies requiring a reinforcement of the therapeutic arsenal. It is likely that it is in the poorest countries or countries where there is no pharmaceutical industry, and where there are no conflicts of interest with new molecules, that clinical trials involving old molecules will develop, which may be extremely profitable. This is already the case in Pakistan, Iraq, and Bangladesh where clinical trials evaluating the efficacy of such molecules in COVID-19 are already taking place (https://clinicaltrials.gov). In any case, we are experiencing a turning point in infectious disease therapeutics, given that there are a considerable number of molecules of natural origin or produced or improved by humans, which have multiple activities and have the advantage of being both inexpensive and having a level of toxicity that is perfectly known and identified, which can save several years and considerable sums of money to achieve for entirely new products. However, the economic model allowing molecules of this nature to be repositioned is currently lacking in Western countries and will have to be the subject of major political reflection.

## Future perspective

*In vitro* sensitivity assays on cell culture are the basis and fundamental proof of concept for the development of novel and repurposed drugs, including for COVID-19 therapeutics. Drugs with proven efficiency in preclinical studies are the most promising and hence of utmost priority in testing in clinical studies. In the specific context of health emergency, clinical studies focusing on the repurposed drugs with a well-known safety profile and for which a certain degree of evidence of activity that has been shown *in vitro* could be prioritized. High-throughput *in vitro* sensitivity screens in cell cultures allow a quick identification of many compounds that will be suitable treatment candidates for further evaluation in clinical trials. *In vitro* sensitivity assays are a prerequisite for coordinated, well designed and useful clinical trials. Moreover, as the antiviral therapy requires an early administration in the course of the in COVID-19, best drug candidates should have simple use, possibility of self administration and widespread availability, after an early diagnosis of SARS-CoV-2 infection. Finally, as all the attention is currently focused on vaccines as the one and only means of combating the virus, we may have to wait until we have better identified the failures or at least the flaws of the vaccine strategy to be able to relaunch work on the interest of antiviral molecules. This should be considered as a lesson learned from this pandemic in the aim of not wasting time in treating patients. Moreover, repurposed drugs used for the treatment of newly emerging pathogens could guarantee an easier global worldwide access, including developing countries that may not benefit new expensive developed drug. Drug repurposing is a different view of pharmaceutical research, and it undoubtedly marks a turning point in infectious disease therapeutics and deserves to be the subject of major political reflection.

**Executive summary**

**Background**

- Drug repurposing represents the most pertinent strategy in the context of a pandemic, due to the health emergency, the low cost of development and a known safety profile.

**Materials & Methods**

- Review of the literature was carried out on PubMed, Google Scholar, and completed by data extracted from the Stanford coronavirus antiviral research database as on 25 July 2021 and included both cell culture assays and molecular docking studies.
- We noted a great lack of standardization in *in vitro* sensitivity cell culture assays, according to the studies.

**Results**

**Antivirals**

- Several RdRp inhibitors showed an *in vitro* activity against SARS-CoV-2. Small clinical studies confirmed a possible benefit for only two of these: favipiravir and the combination sofosbuvir + daclatasvir.

**Antiparasitic agents**

- Chloroquine and hydroxychloroquine exhibit an unambiguous anti-SARS-CoV-2 activity in cell cultures with clinical contradictory results.
- Nitazoxanide inhibits SARS-CoV-2 replication *in vitro* at micromolar concentrations, together with a starting evidence of clinical benefit for mild respiratory insufficiency due to SARS-COV-2 infection both on the duration of hospital stay and on the viral clearance.
- Ivermectin showed an anti-SARS-CoV-2 activity congruently with clinical evidence on a reduced risk of death if used as a treatment of COVID-19 and on a reduced risk of SARS-CoV-2 infection if used as a prophylaxis.

**Antibiotics**

- Clofazimine, azithromycin, doxycycline and clofoctol exhibit substantial anti-SARS-CoV-2 activity *in vitro*.
- Interestingly, clofoctol lowers viral load and inflammation in lung of mice experimentally infected by SARS-CoV-2.

**Antipsychotics**

- With variable antiviral activity against HCoVs, they do not represent a therapeutic option due to their inherent pharmacological properties.

**Antihistaminics**

- Desloratadine and ebastine exhibit an anti-SARS-CoV-2 activity in culture cells at micromolar concentrations.

**Kinase inhibitors**

- Several kinase inhibitors show a potent activity against SARS-CoV-2 at submicromolar concentrations, but the benefit/risk profile for their use in COVID-19 may not be favorable.

**Immunosuppressive agents**

- For organ transplant recipients chronically treated by these compounds, these molecules show an interesting anti-SARS-CoV-2 activity at very low concentrations.

**Cardiac glycosides**

- Although they cannot reasonably be an option for the treatment of COVID-19, several sensitivity assays have concluded their anti-SARS-CoV-2 activity.

**Antineoplastics**

- Their potent antiviral activity against SARS-CoV, SARS-CoV-2 or MERS-CoV according to the compounds, shown in a few *in vitro* studies, could be interesting to explore in patients currently treated by these molecules.

**Anti-estrogens**

- Non-steroidal, anti-estrogen compounds indicated in the treatment of breast cancers have activities against SARS-CoV-2, which are very variable according to the studies.

**Calcium channel blocker with an action on the cardiovascular system**

- Verapamil used for hypertension inhibits SARS-CoV-2-infected Huh7 cells at low concentrations.

**Drugs acting on the alimentary tract & metabolism**

- Metoclopramide showed on Huh7 cells, an anti-SARS-CoV-2 activity at a MOI of 0.2 with an EC_50_ of 0.5 μM.

**Glycosaminoglycan attachment inhibitors**

- Heparin harbored an *in vitro* anti-SARS-CoV-2 activity at therapeutical concentrations and has been associated with lower mortality in patients even when the saturation of oxygen was <90%.

**Others**

- Although antifibrinolytics showed a potential anti-SARS-CoV-2 activity especially for camostat and nafamostat, they cannot be considered as therapeutic options, given the thrombotic risk in COVID-19.
- Efficacy of camostat has anyway been assessed in early stages of infection of COVID-19, but it did not show any clinical benefit, but notably without any serious adverse event.
- Auranofin also induces a great reduction in viral SARS-CoV-2 RNA at 48 h, in cell cultures, at a low concentration and also harbors the advantages of anti-inflammatory properties.

**Conclusion**

- Finally, when comparing the results of molecular docking with those of *in vitro* sensitivity assays and results from the first clinical studies, and, when considering the possibility of oral administration, the global safety profile, 11 molecules may appear as therapeutic options in COVID-19: azithromycin, clofazimine, clofoctol, nelfinavir, favipiravir, ivermectin, nitazoxanide, amodiaquine, heparin, chloroquine and hydroxychloroquine.
- These molecules are known for a long time. All of them are available and are of extremely low cost, which is a turning point in infectious disease therapeutics, given the considerable number of old molecules with multiple activities. This offers the advantage of being inexpensive and having a known safety profile, at the opposite of new molecules that furthermore need long-time development non compatible with this context of health emergency.
