# Supplementary material for: Drug repurposing against SARS-CoV-1, SARS-CoV-2 and MERS-CoV
Source: Future Microbiol. 2021 Nov 10:10.2217/fmb-2021-0019. doi: 10.2217/fmb-2021-0019 (PMC8579950; doi:10.2217/fmb-2021-0019)
Supplement: Supplementary file 1 [file SUPPLEMENTARY_TABLE1.docx]

**SUPPLEMENTARY TABLE 1**

**List of druggable compounds for which a molecular docking study predicted an interaction with a viral protein of SARS-CoV-2.**

| Abacavir | Li et al preprint 2020 (doi:10.20944/preprints202003.0286.v1) |
| --- | --- |
| Acarbose | Prajapat, J Mol Graph Model, 2020 |
| Adalimumab | Li et al preprint 2020 (doi:10.20944/preprints202003.0286.v1) |
| Afelimomab | Li et al preprint 2020 (doi:10.20944/preprints202003.0286.v1) |
| Aliskiren | Eleftheriou, Molecules, 2020 |
| Alprazolam | Gimeno, Int J Mol Sci 2020 |
| Amentoflavone | Chitranshi, J Transl Med. 2020 |
| Amikacin | Prajapat, J Mol Graph Model, 2020 |
| Aminoquinoline | El-hoshoudy, J Mol Liquids 2020 |
| Amodiaquine | Peele, Inform Med Unlocked. 2020 |
| amrubicin | Jiménez-Alberto |
| Anagliptin | Eleftheriou, Molecules, 2020 |
| Andrographolide | Li et al preprint 2020 (doi:10.20944/preprints202003.0286.v1) |
| Anidulafungin | Anwar ChemXriv (pre-print) 2020 |
| Antrafenine | Wei, Chin J Integr Med 2020 |
| Apigenin | Chitranshi, J Transl Med. 2020 |
| Argatroban | Eleftheriou, Molecules, 2020 |
| Artemisinin | Chitranshi, J Transl Med. 2020 |
| Artenimol | Li et al preprint 2020 (doi:10.20944/preprints202003.0286.v1) |
| Atiprimod | Li et al preprint 2020 (doi:10.20944/preprints202003.0286.v1) |
| Azithromycin | El-hoshoudy, J Mol Liquids 2020; Fantini, Int J Antimicrob Agents, 2020 ; Bezerra Braz, International Journal of Antimicrobial Agents, 2020 |
| Bamifyllin | Arun, Journal of Biomolecular Structure and Dynamics 2020 |
| Betrixaban | Eleftheriou, Molecules, 2020 |
| Bilobetin | Chitranshi, J Transl Med. 2020 |
| Binifibrate | Arun, Journal of Biomolecular Structure and Dynamics 2020 |
| Birinapant | Lokhande, Journal of Biomolecular Structure and Dynamics 2020 |
| Boceprevir | Eleftheriou, Molecules, 2020 |
| Bolazine b | Chen F1000 Research 2020 |
| Brecanavir | Hakmi, Bioinformation, 2020 |
| Bromocriptine | Gul, J Biomol Struct Dyn 2020 |
| Cangrelor | Prajapat, J Mol Graph Model, 2020 |
| Captopril | Eleftheriou, Molecules, 2020 |
| Carmofur | El-hoshoudy, J Mol Liquids 2020 |
| Carprofen | Gimeno, Int J Mol Sci 2020 |
| Cefazolin | Li et al preprint 2020 (doi:10.20944/preprints202003.0286.v1) |
| Cefuroxime | Elfiky, Journal of Biomolecular Structure and Dynamics 2020 |
| Celecoxib | Gimeno, Int J Mol Sci 2020 |
| Cepharanthine | Ruan J Med Virol 2020 |
| Cetrorelix | Pokhrel, J Med Microbiol 2020 |
| Chloroquine | Li et al (preprint) 2020; El-hoshoudy, J Mol Liquids 2020; Bezerra Braz, International Journal of Antimicrobial Agents, 2020; Chitranshi, J Transl Med. 2020 |
| Ciluprevir | Hakmi, Bioinformation, 2020 |
| Cinanserin | El-hoshoudy, J Mol Liquids 2020 |
| cinoxacin | Baby, F1000 Research, 2020 |
| ciprofloxacin | Br, F1000 Research, 2020 |
| Clenbuterol | Li et al preprint 2020 (doi:10.20944/preprints202003.0286.v1) |
| Clofazimine | Hosseini Life Sciences 2020 |
| Cobicistat | Ibrahim, J Biomol Struct Dyn 2020 |
| conivaptan | Gul, J Biomol Struct Dyn 2020 |
| CyclosporineA | El-hoshoudy, J Mol Liquids 2020 |
| Dabigatran | Eleftheriou, Molecules, 2020 |
| Dactinomycin | Pokhrel, J Med Microbiol 2020 |
| Danoprevir | Eleftheriou, Molecules, 2020 |
| Darunavir | Chitranshi, J Transl Med. 2020 |
| Daunorubicin | Jiménez-Alberto |
| Deldeprevir | Hakmi, Bioinformation, 2020 |
| Desmopressin | Anwar ChemXriv (pre-print) 2020 |
| Didanosine | Cava, Viruses, 2020 |
| Diflunisal | Baby, F1000 Research, 2020 |
| Digitoxin | Wei, Chin J Integr Med 2020 |
| Dihydroergocristine a | Chen F1000 Research 2020 |
| Dihydroergotamine | Gul, J Biomol Struct Dyn 2020 |
| Diosmin a b | Chen F1000 Research 2020 |
| Disulfiram | El-hoshoudy, J Mol Liquids 2020 |
| Ditercalinium | Chen F1000 Research 2020 |
| Doxycycline | Sachdeva, OMICS 2020 |
| Dutasteride | Gul, J Biomol Struct Dyn 2020 |
| Dynasore | Lokhande, Journal of Biomolecular Structure and Dynamics 2020 |
| Ebselen | El-hoshoudy, J Mol Liquids 2020 |
| Edoxaban | Anwar ChemXriv (pre-print) 2020; Eleftheriou, Molecules, 2020 |
| Elbasvir | Anwar ChemXriv (pre-print) 2020; Behloul, Eur J Pharmacol, 2020 |
| Eluxadoline a b | Chen F1000 Research 2020 |
| Elvitegravir | Alexpandi, Front Microbiol. 2020 |
| Emetine | Das Journal of Biomolecular Structure and Dynamics 2020; |
| Emodin | El-hoshoudy, J Mol Liquids 2020 |
| enasidenib | Anwar ChemXriv (pre-print) 2020 |
| Entrectinib | Wei, Chin J Integr Med 2020 |
| Epinephrine | Li et al preprint 2020 (doi:10.20944/preprints202003.0286.v1) |
| Epirubicin | Khan, Journal of Biomolecular Structure and Dynamics 2020; |
| ergotamine | Gul, J Biomol Struct Dyn 2020 |
| Etanercept | Li et al preprint 2020 (doi:10.20944/preprints202003.0286.v1) |
| ethyl biscoumacetate | Gimeno, Int J Mol Sci 2020 |
| etodolac | Baby, F1000 Research, 2020 |
| Etoposide a b | Chen F1000 Research 2020 |
| Everolimus | Kadioglu (https://www.who.int/bulletin/online_first/20-255943.pdf) |
| Evogliptin | Eleftheriou, Molecules, 2020 |
| Faldaprevir | Hakmi, Bioinformation, 2020; Eleftheriou, Molecules, 2020 |
| Favipiravir | El-hoshoudy, J Mol Liquids 2020; Sada Microorganisms 2020; Chitranshi, J Transl Med. 2020 |
| Fenoterol | Prajapat, J Mol Graph Model, 2020 |
| Filibuvir | Ruan J Med Virol 2020 |
| Flavin adenine dinucleotide | Wei, Chin J Integr Med 2020 |
| Flunitrazepam | Wei, Chin J Integr Med 2020 |
| Flutamide | Cava, Viruses, 2020 |
| FluticasonePropionate | Cava, Viruses, 2020 |
| fluvastatin | Baby, F1000 Research, 2020 |
| fospropofol | Baby, F1000 Research, 2020 |
| Framycetin | Li et al preprint 2020 (doi:10.20944/preprints202003.0286.v1) |
| Galidesivir | Elfiky, Life Sciences 2020; Chitranshi, J Transl Med. 2020 |
| Gemigliptin | Eleftheriou, Molecules, 2020 |
| Ginkgetin | Chitranshi, J Transl Med. 2020 |
| Glecaprevir | Anwar ChemXriv (pre-print) 2020; Shamsi, Biosci Rep 2020 |
| Glycyrrhizic acid | Br, F1000 Research, 2020 |
| Glycyrrhizinate | Jiménez-Alberto |
| Golimumab | Li et al preprint 2020 (doi:10.20944/preprints202003.0286.v1) |
| Gosogliptin | Eleftheriou, Molecules, 2020 |
| Grazoprevir | Hakmi, Bioinformation, 2020; Kadioglu (https://www.who.int/bulletin/online_first/20-255943.pdf); Behloul, Eur J Pharmacol, 2020 |
| Heparan sulfate | Kim, Antiviral Res 2020 |
| Hesperidin | Das Journal of Biomolecular Structure and Dynamics 2020; |
| Hesperidin a d | Chen F1000 Research 2020 |
| Hydroxychloroquine | El-hoshoudy, J Mol Liquids 2020; Bezerra Braz, International Journal of Antimicrobial Agents, 2020; Chitranshi, J Transl Med. 2020; Fantini, Int J Antimicrob Agents, 2020 |
| Ibalizumab | Li et al preprint 2020 (doi:10.20944/preprints202003.0286.v1) |
| Indinavir | Das Journal of Biomolecular Structure and Dynamics 2020; Hall Travel Medicine and Infectious Disease 2020; Hakmi, Bioinformation, 2020 |
| Indomethacinhave | Jiménez-Alberto |
| Infliximab | Li et al preprint 2020 (doi:10.20944/preprints202003.0286.v1) |
| Inogatran | Eleftheriou, Molecules, 2020 |
| Irinotecan a b | Chen F1000 Research 2020 |
| Ivermectin | Kadioglu (https://www.who.int/bulletin/online_first/20-255943.pdf) |
| Kanamycin | Prajapat, J Mol Graph Model, 2020 |
| Kappadione | Baby, F1000 Research, 2020 |
| Ketoprofen | Li et al preprint 2020 (doi:10.20944/preprints202003.0286.v1) |
| Lamivudine | Prajapat, J Mol Graph Model, 2020 |
| Ledipasvir | Kadioglu (https://www.who.int/bulletin/online_first/20-255943.pdf) |
| Ledipasvir a | Chen F1000 Research 2020 |
| Lemborexant | Wei, Chin J Integr Med 2020 |
| Leucovorin | Lokhande, Journal of Biomolecular Structure and Dynamics 2020 |
| levomefolic acid | Prajapat, J Mol Graph Model, 2020; Baby, F1000 Research, 2020 |
| Linagliptin | Eleftheriou, Molecules, 2020 |
| Lonafarnib | Ruan J Med Virol 2020 |
| Lopinavir | Das Journal of Biomolecular Structure and Dynamics 2020; Hakmi, Bioinformation, 2020; El-hoshoudy, J Mol Liquids 2020; Eleftheriou, Molecules, 2020; Chitranshi, J Transl Med. 2020; Peele, Inform Med Unlocked. 2020 |
| Loxapine | Wei, Chin J Integr Med 2020 |
| Lumacaftor a | Chen F1000 Research 2020 |
| Luteolin | Chitranshi, J Transl Med. 2020 |
| Maraviroc | Shamsi, Biosci Rep 2020; Li et al preprint 2020 (doi:10.20944/preprints202003.0286.v1) |
| Meclocycline | Jiménez-Alberto |
| Mefenamic acid | Baby, F1000 Research, 2020 |
| Melagatran | Eleftheriou, Molecules, 2020 |
| Melogliptin | Eleftheriou, Molecules, 2020 |
| Mitoxantrone | Lokhande, Journal of Biomolecular Structure and Dynamics 2020; Prajapat, J Mol Graph Model, 2020 |
| Mizolastine | Hosseini Life Sciences 2020 |
| MK-3207 c | Chen F1000 Research 2020 |
| Montelukast | Baby, F1000 Research, 2020 |
| Myricetin | Li et al preprint 2020 (doi:10.20944/preprints202003.0286.v1) |
| Nelfinavir | Musarrat, J Med Virol, 2020; Huynh, J Phys Chem Lett, 2020 |
| N-Formylmethionine | Li et al preprint 2020 (doi:10.20944/preprints202003.0286.v1) |
| Nicotinamide | Kandeel, Life Sci, 2020 |
| Nilotinib | Ruan J Med Virol 2020; Wei, Chin J Integr Med 2020 |
| Nimesulide | Cava, Viruses, 2020 |
| Norfloxacin | Baby, F1000 Research, 2020 |
| Nystatin | Kadioglu (https://www.who.int/bulletin/online_first/20-255943.pdf) |
| Olsalazine | Li et al preprint 2020 (doi:10.20944/preprints202003.0286.v1) |
| Olysio | Ruan J Med Virol 2020 |
| Omarigliptin | Eleftheriou, Molecules, 2020 |
| Oxatomide | Anwar ChemXriv (pre-print) 2020 |
| Oxolinicacid | Alexpandi, Front Microbiol. 2020 |
| Paliperidone | Gul, J Biomol Struct Dyn 2020 |
| Paritaprevir | Hakmi, Bioinformation, 2020; Kadioglu (https://www.who.int/bulletin/online_first/20-255943.pdf) |
| Pazopanib | Wei, Chin J Integr Med 2020 |
| Penicillin G | Anwar ChemXriv (pre-print) 2020 |
| Perampanel | Gimeno, Int J Mol Sci 2020 |
| Photofrin | Cava, Viruses, 2020 |
| Pitavastatin | Baby, F1000 Research, 2020 |
| Pranlukast | Li et al preprint 2020 (doi:10.20944/preprints202003.0286.v1) |
| Procaine | Anwar ChemXriv (pre-print) 2020 |
| Proline | Li et al preprint 2020 (doi:10.20944/preprints202003.0286.v1) |
| Pseudoephedrine | Li et al preprint 2020 (doi:10.20944/preprints202003.0286.v1) |
| Pyronaridine | Hosseini Life Sciences 2020 |
| Quercetin | Li et al preprint 2020 (doi:10.20944/preprints202003.0286.v1); Chitranshi, J Transl Med. 2020 |
| Quinupristin | Pokhrel, J Med Microbiol 2020 |
| R428 b | Chen F1000 Research 2020 |
| Raltegravir | Wei, Chin J Integr Med 2020 |
| Remdesivir | Hall Travel Medicine and Infectious Disease 2020; El-hoshoudy, J Mol Liquids 2020; Chitranshi, J Transl Med. 2020; Elfiky Life Sciences 2020; |
| Ribavirin | El-hoshoudy, J Mol Liquids 2020; Elfiky, Life Sciences 2020; Kandeel, Life Sci, 2020 |
| riboflavin | Prajapat, J Mol Graph Model, 2020 |
| ridogrel | Baby, F1000 Research, 2020 |
| Rifabutin | Kadioglu (https://www.who.int/bulletin/online_first/20-255943.pdf); Anwar ChemXriv (pre-print) 2020 |
| Rifampicin | Pokhrel, J Med Microbiol 2020 |
| Rilapladib | Alexpandi, Front Microbiol. 2020 |
| Ritonavir | Das Journal of Biomolecular Structure and Dynamics 2020; El-hoshoudy, J Mol Liquids 2020; Eleftheriou, Molecules, 2020; Chitranshi, J Transl Med. 2020 |
| Rivaroxaban | Eleftheriou, Molecules, 2020 |
| Rosoxacin | Baby, F1000 Research, 2020 |
| Ruplizumab | Li et al preprint 2020 (doi:10.20944/preprints202003.0286.v1) |
| Rutin | Das Journal of Biomolecular Structure and Dynamics 2020; |
| Saquinavir | Hall Travel Medicine and Infectious Disease 2020; Ruan J Med Virol 2020; Hakmi, Bioinformation, 2020; Khan, Journal of Biomolecular Structure and Dynamics 2020;Alexpandi, Front Microbiol. 2020 |
| Sarafloxacin | Gimeno, Int J Mol Sci 2020 |
| Saxagliptin | Eleftheriou, Molecules, 2020 |
| Selinexor | Anwar ChemXriv (pre-print) 2020 |
| Siltuximab | Li et al preprint 2020 (doi:10.20944/preprints202003.0286.v1) |
| Simeprevir | Hakmi, Bioinformation, 2020; Hosseini Life Sciences 2020; Kadioglu (https://www.who.int/bulletin/online_first/20-255943.pdf) |
| Sirolimus | Pokhrel, J Med Microbiol 2020 |
| Sitagliptin | Eleftheriou, Molecules, 2020 |
| Sofosbuvir | Elfiky Life Sciences 2020 |
| Sovaprevir | Eleftheriou, Molecules, 2020; Behloul, Eur J Pharmacol, 2020 |
| Streptomycin, | Br, F1000 Research, 2020 |
| Tadalafil | Anwar ChemXriv (pre-print) 2020 |
| Tapinarof | Li et al preprint 2020 (doi:10.20944/preprints202003.0286.v1) |
| Tedizolid phosphate | Baby, F1000 Research, 2020 |
| Tegobuvir | Ruan J Med Virol 2020 |
| Teicoplanin | Tripathi, Int J Biol Macromol 2020 |
| Telaprevir | Eleftheriou, Molecules, 2020 |
| Telbivudine | Kandeel, Life Sci, 2020 |
| Teneliglipti | Eleftheriou, Molecules, 2020 |
| Teniposide | Kadioglu (https://www.who.int/bulletin/online_first/20-255943.pdf) |
| Teniposide a b | Chen F1000 Research 2020 |
| Tenofovir | Elfiky, Life Sciences 2020 |
| Terlipressin | Anwar ChemXriv (pre-print) 2020 |
| Thalidomide | Li et al preprint 2020 (doi:10.20944/preprints202003.0286.v1) |
| Theaflavin digallate | Peele, Inform Med Unlocked. 2020 |
| Thiabendazole | Cava, Viruses, 2020 |
| Tideglusib | El-hoshoudy, J Mol Liquids 2020 |
| Tipranavir | Ruan J Med Virol 2020; Gul, J Biomol Struct Dyn 2020 |
| Trelagliptin | Eleftheriou, Molecules, 2020 |
| Tretinoin | Dey, Comput Biol Med, 2020 |
| Trovafloxacin | Gimeno, Int J Mol Sci 2020 |
| UK-432097 c | Chen F1000 Research 2020 |
| Umifenovir | Chitranshi, J Transl Med. 2020 |
| ValrubicinmetaboliteN-trifluoroacetyladriamycin | Jiménez-Alberto |
| Vaniprevir | Eleftheriou, Molecules, 2020 |
| Vapreotida | Khan, Journal of Biomolecular Structure and Dynamics 2020; |
| Velpatasvir | Kadioglu (https://www.who.int/bulletin/online_first/20-255943.pdf); Anwar ChemXriv (pre-print) 2020; Chen F1000 Research 2020 |
| Venetoclax | Chen F1000 Research 2020 |
| Vicriviroc | Li et al preprint 2020 (doi:10.20944/preprints202003.0286.v1) |
| Vidarabine | Prajapat, J Mol Graph Model, 2020 |
| Vildagliptin | Eleftheriou, Molecules, 2020 |
| Viomycin | Mahanta, J Biomol Struct Dyn 2020 |
| Vitamin C | Chitranshi, J Transl Med. 2020 |
| Vitamin D | Chitranshi, J Transl Med. 2020 |
| Vitamin E | Chitranshi, J Transl Med. 2020 |
| vitaminB12 | Kandeel, Life Sci, 2020 |
| YSIL6 | Li et al preprint 2020 (doi:10.20944/preprints202003.0286.v1) |
| Zanamivir | Hall Travel Medicine and Infectious Disease 2020 |
